# Robustness and plasticity in *Drosophila* heat avoidance

**DOI:** 10.1038/s41467-021-22322-w

**Published:** 2021-04-06

**Authors:** José Miguel Simões, Joshua I. Levy, Emanuela E. Zaharieva, Leah T. Vinson, Peixiong Zhao, Michael H. Alpert, William L. Kath, Alessia Para, Marco Gallio

**Affiliations:** 1 Department of Neurobiology, Northwestern University, Evanston, IL USA; 2 Department of Engineering Sciences and Applied Mathematics, Northwestern University, Evanston, IL USA

**Keywords:** Neural circuits, Sensorimotor processing

## Abstract

Simple innate behavior is often described as hard-wired and largely inflexible. Here, we show that the avoidance of hot temperature, a simple innate behavior, contains unexpected plasticity in *Drosophila*. First, we demonstrate that hot receptor neurons of the antenna and their molecular heat sensor, Gr28B.d, are essential for flies to produce escape turns away from heat. High-resolution fly tracking combined with a 3D simulation of the thermal environment shows that, in steep thermal gradients, the direction of escape turns is determined by minute temperature differences between the antennae (0.1°–1 °C). In parallel, live calcium imaging confirms that such small stimuli reliably activate both peripheral thermosensory neurons and central circuits. Next, based on our measurements, we evolve a fly/vehicle model with two symmetrical sensors and motors (a “Braitenberg vehicle”) which closely approximates basic fly thermotaxis. Critical differences between real flies and the hard-wired vehicle reveal that fly heat avoidance involves decision-making, relies on rapid learning, and is robust to new conditions, features generally associated with more complex behavior.

## Abstract (web summary)

Simões, Levy et al. use a combination of experiments and models to study how *Drosophila* flies steer away from dangerous heat. They discover that flies use small temperature differences between the antennae to turn clear of thermal danger; they also demonstrate that heat avoidance, a simple innate behavior, contains unexpected plasticity.

## Introduction

Innate behaviors can be performed in response to a cue without prior experience. As such they are considered hard-wired in the nervous system, particularly in the more “simple” nervous system of invertebrates. At the extreme, the cyberneticist Valentino Braitenberg famously proposed that a broad range of seemingly complex animal behavior (e.g. “aggression”, “love”, and “hate”^1^) could be reproduced by hypothetical vehicles purely as a result of the wiring pattern of a set of symmetric sensors and motors.

Here, we report the first high-resolution characterization of heat avoidance and thermotaxis in adult *Drosophila melanogaster*. Our first goal is to define the basic functional organization of the sensory system that guides these innate behaviors in adult fruit flies. Our next objective is to compare fly heat avoidance with the behavior of a Braitenberg-inspired in silico vehicle model, explicitly probing the notion that the fly’s innate avoidance of hot temperature can be understood as a combination of hard-wired responses.

The avoidance of unfavorable temperatures is a fundamental behavior in the repertoire of all motile animals, from flatworms^2^ to whale sharks^3^. Because of its ancestral nature, heat avoidance is an ideal system to test the idea that simple innate behavior may be largely hard-wired. The fruit fly *Drosophila melanogaster* is an excellent model to address this question. A genetically and physiologically tractable animal capable of elaborate short and long-range navigation^4,5^, flies have been extensively used to study and model the sensory processing that informs navigational decisions^6,7^.

When given a choice, adult *Drosophila melanogaster* prefers 25 °C over colder or warmer temperatures^8,9^. Flies are, in particular, very sensitive to heat: in laboratory conditions, adults of both sexes are incapacitated if confined to ~35–37 °C^10^, and exposure to 40 °C proves quickly lethal. As a consequence, adult flies display increasingly robust avoidance of temperatures higher than their preferred 25 °C, spanning the innocuous (i.e. not harmful, 25–35 °C) and noxious (i.e. potentially harmful or even lethal, >35 °C) range.

**Fig. 1:**
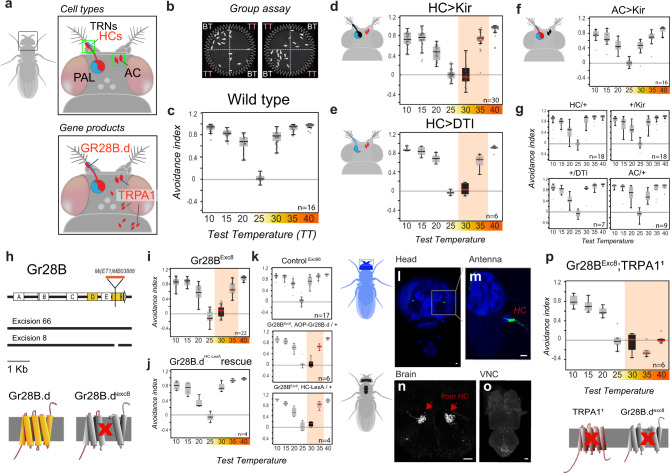
Noxious and innocuous heat sensing together mediate heat avoidance in *Drosophila*. **a** Schematic representation of the cell types and gene products involved in heat sensing in adult *Drosophila* (TRNs: temperature receptor neurons, HCs: hot cells, AC: anterior cells, PAL: posterior antennal lobe). **b**, **c** Two-choice assay for temperature preference. **b** Groups of flies are given a choice between a base temperature (BT, 25 °C; *n* = no. of groups) and a variable test temperature (TT; a single video frame is shown). **c** Temperature preference is quantified as an avoidance index for the various test temperatures (wild type is shown). **d** Genetic silencing (by expression of Kir2.1, a hyperpolarizing agent) or (**e**) ablation (by Diphteria toxin, DTI, a cell killing toxin—under the control of HC-Gal4) of hot TRNs of the arista abolishes avoidance of 30 °C and reduces avoidance of 35 °C. **f** Genetic silencing of AC (in AC-Gal4>UAS-Kir2.1) has no effect on avoidance. **g** Control genotypes (drivers and effectors). **h** Creation of a GR28B null mutant. Schematics of the Gr28B genomic locus, Minos insertion, genomic excisions produced for this work and effect of excisions on the predicted protein. **i** An excision in one of the common exons (Exc8) abolishes avoidance of 30 °C and reduces avoidance of 35 °C. **j** Targeting expression of a GR28B.d cDNA to hot-activated TRNs (by HC-LexA) completely rescues avoidance defects. **k** Controls. **l**–**o** HC-LexA expression visualized by GFP (in HC-LexA>Aop-GFP animals). **l**, **m** Confocal stacks from head/antennae (blue = cuticle autofluorescence, green = GFP expression in Hot TRNs of the arista; scalebars = 20 μm). **n**–**o** 2-photon stacks of brain and ventral nerve chord (VNC), showing (**n**) hot TRNs axon terminals in the brain, and (**o**) no labeling in the VNC (scalebars, 20 µm). **p** *Gr28B*^*Exc8*^*, TRPA1*^*1*^ double mutants display no heat avoidance, but normal cold avoidance. In all boxplots, the edges of the boxes are the first and third quartiles, a solid line marks the median, and whiskers delimit the data range; a solid red median line denotes a significant interaction between experimental and control animals (two-way ANOVA, *P* < 0.001), a black box denotes avoidance indexes not significantly different from zero (one sample t test, *P* < 0.05). See Supplementary Fig. 1 for additional controls. *P*_HC-Kir30_ = 3.78e−12, *P*_HC-Kir35_ = 6.74e−4; *P*_HC-DTI30_ = 1.73e−7, *P*_HC-DTI35_ = 2.74e−5; *P*_HC,AC-Kir30_ = 1.39e−11, *P*_HC,AC-Kir35_ = 4.67e–6; *P*_Exc8-30_ = 8.12e–18, *P*_Exc8-35_ = 3.8e–7; *P*_Exc8AOP-30_ = 5.06e–9; *P*_Exc8AOP-35_ = 8.01e–6; *P*_Exc8lexA-30_ = 9.59e−8; *P*_Exc8lexA-35_ = 6.28e−3; *P*_Exc8Df-30_ = 4.22e−12, *P*_Exc8Df-35_ = 4.14e−6; *P*_GR28TRPA1-30_ = 2.42e−18, *P*_GR28TRPA1-35_ = 1.87e−27, *P*_GR28TRPA1-40_ = 1.95e−25, *P*_HC-Kir30_ = 0.44; *P*_HC-DTI30_ = 0.25; *P*_Exc8-30_ = 0.06; *P*_Exc8Df-30_ = 0.24; *P*_GR28TRPA1-30_ = 0.50, *P*_GR28TRPA1-40_ = 0.80.

In the fly nervous system, rapid temperature changes are detected by dedicated populations of hot- and cold-activated temperature receptor neurons (TRNs) residing in the last antennal segment, the arista^8^. The projections of these neurons form two distinct, adjacent glomeruli in the posterior antennal lobe (PAL) region of the brain, where afferent activity defines a simple map for temperature representation^8,11^. In addition to the hot-activated receptors of the antenna (“Hot Cells” or HC), adult flies possess internal heat sensors within the head capsule (“Anterior Cells” or AC^12^), and multi-modal thermal/mechanical nociceptors innervate the body of both the larva and adult^13,14^ (Fig. 1a shows a schematic of the cell types and gene functions involved in heat sensing in the adult).

Despite this basic knowledge, we still do not understand how the activity of distinct cellular sensors may be coordinated to produce heat avoidance, nor how aversive heat responses become integrated in the navigational programs that steer animals away from thermal danger (thermotaxis).

## Results

To better understand the relative contribution of noxious and innocuous heat responses to heat avoidance and thermotaxis, we first probed the role of the known cellular and molecular pathways for heat detection. For this, we systematically tested a number of genetic and cellular manipulations in a 2-choice temperature preference assay performed on small groups of adult flies^8^ (Fig. 1).

Our results directly support the notion that both noxious and innocuous signals play a role during rapid heat avoidance:

1. Transgenic silencing or ablation of heat-activated TRNs of the arista abolished avoidance of 30 °C heat, while avoidance of 35 °C was only partially reduced, and avoidance of 40 °C was not affected (Fig. 1a–e; note that silencing of AC neurons had no effect on this behavior, Fig. 1f, see Fig. 1g and Supplementary Fig. 1 for controls).
  Identical results to the silencing of hot cells were obtained from a null mutant (produced for this study) of the candidate heat receptor ion channel Gr28B^15^. Importantly, the *Gr28B* mutant phenotype was completely rescued by expression of the Gr28B.d protein variant exclusively in arista hot cells (under the control of a selective HC-LexA line, Fig. 1h–k**;** and see Fig. 1l–o for this driver’s expression pattern).
  Together, these results demonstrate an essential role for the hot-activated TRNs of the arista in the avoidance of innocuous heat and suggest that Gr28B (and Gr28B.d in particular) functions as the main heat receptor for this cell type^15^. We note that this conclusion is in line with earlier results^8,15^ but stands in contrast to a recent report that challenged the role of Gr28B in heat avoidance^16^. This discrepancy is likely due to the fact that a null Gr28B mutant was not available prior to this work.
2. In the adult, the broadly conserved nociceptor TRPA1 mediates noxious heat responses by sensing H_2_O_2_/ROS produced as a result of heat damage^17^. As such, a prominent effect of TRPA1 loss is a stark reduction of heat avoidance in the noxious range (≥35 °C^17^). Strikingly, a *Gr28B-TRPA1* double-mutant completely eliminated heat avoidance, including to 40 °C, a temperature that can be quickly lethal to *D. melanogaster* (Fig. 1p).

We conclude that, while the arista heat-activated TRNs play a dominant role in the avoidance of innocuous heat (<35 °C), the response to noxious temperature engages an additional cellular system—distinct from AC neurons, that uses TRPA1 as the main transducer.

It is worth noting that all of the genetic and cellular manipulations affecting heat sensing—even the apparently entirely heat-insensitive *Gr28B-TRPA1* double mutants- retained normal temperature preference in the “cold” range (i.e. below the preferred temperature of 25 °C, Fig. 1). This evidence again shows that the processing of temperature preference can function quite independently in the hot *vs*. cold range^8,18–20^.

Our results so far demonstrate how both noxious and innocuous signals play a role during rapid heat avoidance, but do not help explain how they may guide the rapid navigational decisions that determine thermotaxis.

**Fig. 2:**
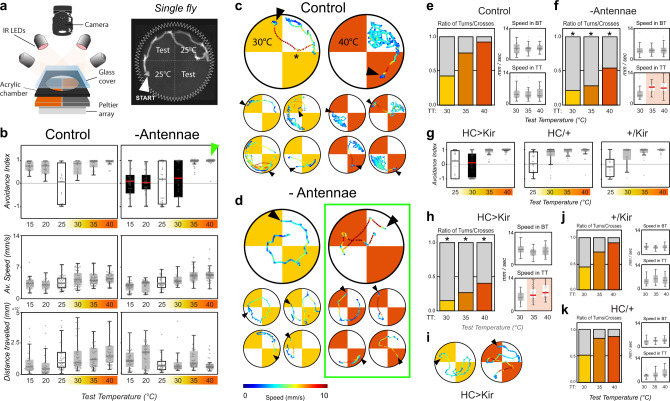
Thermosensory neurons of the arista guide rapid navigation during thermotaxis. **a** Schematic representation of the single fly 2-choice assay for temperature preference. (Test = test temperature). **b** Avoidance indexes and locomotory parameters of (left) single wild-type control flies and (right) single flies in which the antennae had been surgically removed at eclosion. (Top row) Avoidance indexes. Antenna ablated flies display no avoidance for test temperatures ranging from 15 to 30 °C (a solid red median line denotes a significant difference between experimental and controls, ANOVA: *P*_15_ = 1.1e−7, *P*_20_ = 3.7e−6, *P*_30_ = 2.3e−2; a black box denotes avoidance indexes not different from zero, one-sample t test, *P* > 0.05). (Center and Bottom row) Quantification of locomotor parameters shows that antenna ablation does not produce major defects in motility (WT: *N*_15_ = 27, *N*_20_ = 26, *N*_25_ = 43, *N*_30_ = 55, *N*_35_ = 53, *N*_40_ = 55, Ablated: *N*_15_ = 43, *N*_20_ = 36, *N*_25_ = 32, *N*_30_ = 26, *N*_35_ = 38, *N*_40_ = 38). **c**, **d** Single representative tracks from control and antenna-ablated flies. **c** Control flies avoid hot quadrants by producing sharp U-turns at cool/hot boundaries (asterisk; note that in all panels arrowheads denote the position of each fly at the start of heating, and that tracks are color-coded by speed). **d** Antenna ablated flies fail to systematically produce sharp U-turns and instead frequently invade the hot quadrants. **e**, **f** Quantification of the ratio of U-turns/border crosses at the cool/hot boundaries and associated locomotor parameters (Ns as in (**b**)). **e** In control flies the ratio of U-turns/border crosses increases as a function of the temperature on the hot side, until (for test temperature = 40 °C) most border interactions result in U-turns. **f** Antenna-ablated flies display significantly smaller fractions of U-turns at the border in all conditions, but instead display a higher speed for traversals of the 35 °C and 40 °C hot quadrants (highlighted in the lower right panel in (**f**), BT base temperature, TT test temperature). **g**–**i** Genetic silencing of hot-activated TRNs of the arista results in phenotypes in the hot range very similar to antenna ablation (HC/Kir: *N*_25_ = 29, *N*_30_ = 33, *N*_35_ = 36, *N*_40_ = 46, HC/+: *N*_25_ = 27, *N*_30_ = 22, *N*_35_ = 29, *N*_40_ = 26, Kir/+: *N*_25_ = 35, *N*_30_ = 32, *N*_35_ = 26, *N*_40_ = 26; 2-way ANOVA: *P*_30_ = 1.3e−3). **j**, **k** Control genotypes (drivers and effectors). In all boxplots, the edges of the boxes are the first and third quartiles, a solid line marks the median, and whiskers delimit the data range; In (**f**–**k**), a solid red median line denotes a significant interaction between experimental and control animals, a black box denotes avoidance indexes not significantly different from zero. Asterisks in (**f**) and (**h**) denote significant differences in turn/cross ratios from the appropriate controls (asterisk in (**f**): GLMM, Wald test: vs Control *P*_30_ = 3.3e−2, *P*_35_ = 6.4e−11, *P*_40_ = 6.5e−6; red line in (**f**): ANOVA, *P*_35_ = 2.1e−5, *P*_40_ = 5.1e−6, control (**e**); asterisks in (**h**): 2-Way GLMM, Wald test: *P*_30_ = 2.3e−3, *P*_35_ = 1.6e−6, *P*_40_ = 3.0e−2; red line in (**h**): 2-way ANOVA, *P*_35_ = 3.6e−2, *P*_40_ = 1.2e−3, controls (**j**, **k**)).

To get at this question, we modified our assay by adding higher spatial and temporal (30 Hz) resolution to our recording system and tracked the trajectories of individual flies as they navigate between the base (25 °C) and test temperatures in the two-choice arena (Fig. 2a).

This single-fly assay recorded robust avoidance of both hot and cold temperatures (Fig. 2b), with avoidance scores comparable to the ones obtained in the group assays. As expected, and consistent with the fact that the antennae contain both hot and cold receptors^8,21^, ablation of both antennae resulted in a complete loss of avoidance both for temperatures below the preferred 25 °C (the “cold” range: 15 °C, 20 °C) and above it (30 °C; Fig. 2b).

Strikingly, ablation of both antennae did not reduce noxious heat avoidance (Fig. 2b; 35 °C, 40 °C, green arrowhead). However, tracking of single flies revealed that control and antenna-ablated flies used significantly different strategies to achieve heat avoidance in the noxious temperature range.

Whenever control animals encounter the cool/hot boundary within the arena, they perform sharp turns (“U-turns”) and immediately return to the 25 °C quadrant. This behavior is frequently observed at the 25 °C/30 °C boundary (Fig. 2c, asterisk; -and see Fig. 2e for quantification), and becomes prevalent at the 25 °C/40 °C one, such that control flies appear completely confined to the 25 °C quadrant as if contained by an invisible border wall.

Antenna-ablated flies appear unable to efficiently produce such U-turns, and instead often invade the hot quadrants (Fig. 2d, f). Interestingly, while this failure to turn away appears to have little consequence in the 25 °C/30 °C condition (resulting in no avoidance for 30 °C), invasion of the 35 °C or 40 °C quadrants produces faster movement (Fig. 2d, f), which ultimately results in escape from heat and in an overall high avoidance index for the noxious temperatures (Fig. 2b).

The differential effect on the avoidance of 30 °C vs 35 °C and 40 °C is reminiscent of what was observed when silencing heat-activated TRNs of the arista (see Fig. 1). Indeed, when subjected to this single-fly assay, animals in which heat-activated TRNs had been genetically silenced displayed a remarkably similar phenotype to that of antenna-ablated flies: no avoidance for 30 °C but high avoidance for 35 °C and 40 °C, a reduced fraction of U-turns at the 25 °C/hot borders, accompanied by increased speed in the hot quadrants (Fig. 2g–i, see Fig. 2j, k for controls).

Taken together, our results suggest that the heat-activated TRNs of the arista play a key role in the avoidance of both noxious and innocuous heat, by allowing flies to produce sharp turns away from hot boundaries to efficiently escape both aversive and dangerous conditions. In the absence of the antennae (or when signaling from the heat-activated TRNs is impaired), flies are unable to effectively produce U-turns away from heat, but while they appear indifferent to innocuous conditions (30 °C), they react to noxious heat by increasing their speed.

The fact that this increase in speed does not require the antenna (Fig. 2), together with our results on *Gr28B-TRPA1* double mutants (which appear completely insensitive to both noxious and innocuous heat, Fig. 1), suggests that this effect is likely mediated by TRPA1-expressing nociceptors in the fly head and/or body.

Hence, heat-activated TRNs of the arista are essential to control the thermotactic responses that allow flies to efficiently steer away from aversive and potentially dangerous heat. But how are the signals from TRNs used to chart a trajectory that quickly puts the fly out of harm’s way? To address this question, we next reconstructed the profile of the temperature gradient that flies encounter when crossing the border between cool (25 °C) and hot quadrants.

**Fig. 3:**
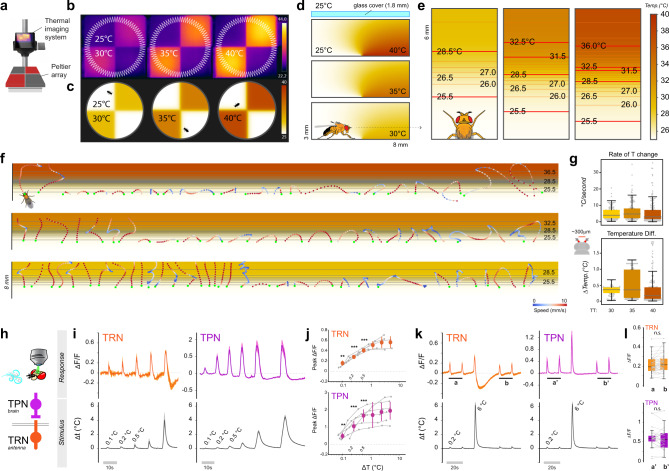
A three-dimensional simulation of the thermal environment reveals small temperature differences are salient stimuli. **a** Schematic representation of the thermal imaging system. **b** Thermal images of the arena in the three experimental conditions, and (**c**), at the same scale, thermal conditions predicted by the simulation (see scale bars for temperature). **d** Side view of a 3 x 8mm section of the experimental chamber, centered on the interface between floor tiles set at 25°/30°, 25°/35°, and 25°/40 °C, respectively, and showing the predicted thermal conditions (note that the glass cover on top is not to scale). **e** Top view of the simulated thermal gradients the fly encounters at the cool/hot boundary, produced by slicing the 3D model at the height of the antennae (~700 μm; note that the 3 panels are not aligned; scalebar: temperature in °C). **f** Representative fly trajectories overlaid atop the gradients in (**e**). Tracks are color-coded by translational speed (see scalebar). Each dot represents the position of the fly head (acquired at 30 Hz). A green dot indicates the fly head position upon entry in the boundary region. **g** Maximum rate of temperature change (top) and maximum inter-antennal temperature difference (bottom) experienced by flies traversing the border in the three experimental conditions. **h** Schematic representation of the 2-photon calcium imaging setup and of the cell types targeted for recording (temperature receptor neurons, TRN and second-order projection neurons, TPN). **i** Average stimuli (bottom) and response traces (top) recorded from TRN axon terminals (orange trace, left) and TPN (purple trace, right) each separately targeted by transgenic expression of G-CaMP7f (traces represent average ±STD of *N*_TRN_ = 5 from 5 flies, *N*_TPN_ = 6 from 6 flies). **j** Orange and purple dots, peak fluorescence averages from data in (**i**), ± STD (bin width starting at 0.1 °C and doubling in size for each consecutive bin; asterisk = significantly different from zero, one sample *t* test, *P* < 0.05; *t* test TRN: *P*_0.1_ = 3.5e−3, *P*_0.2_ = 1.5e−6, *P*_0.5_ = 4.1e−4; TPN: *P*_0.1_ = 2.1e−3, *P*_0.2_ = 1.5e−4, *P*_0.5_ = 9.0e−5). **k**, **l** Exposure to a larger heat stimulus does not lead to sensitization to smaller stimuli. **k** Average stimuli (bottom) and responses (top) ± STD. **l** Average peak signal recorded before (**a**, **a**′) and after (**b**, **b′**) a 6 °C stimulus are not different (n.s. not significant; paired *t* tests; (**k**) and (**l**) are from *N*_TRN_ = 18, *N*_TPN_ = 12 from 7 and 6 animals, respectively; note that the twin peaks in (**a**, **a′**, **b**, and **b′**) are considered independently in (**l**)).

First, we used a thermal imaging system equipped with a macro lens to ensure that the border between 25 °C and hot floor tiles was reasonably sharp and homogeneous (Fig. 3a, b). Taking into consideration the physical dimensions of the chamber, the thermal conductivity of air, the heat transfer coefficient of the materials used and the potential impact of convection (see methods for details), we produced a realistic, high-resolution, three-dimensional simulation of temperature distribution within the arena in the various experimental conditions (Fig. 3c, d and Supplementary Fig. 2).

Next, we “sliced” this 3D volume at the height of the fly antenna (~700 µm) and produced two-dimensional models representing the steep thermal gradient that is expected to form between 25 °C and hot floor tiles (Fig. 3e). Finally, we overlaid the dynamic trajectories that flies performed in our assays as a result of encounters with the border (Fig. 3f), and derived the temperature values at each antenna at any given time point during a border interaction (i.e. based on a simple rigid model of the fly body, in which the antennae protrude from the head and are separated by 300 µm).

Our simulation reveals a number of interesting features of the sensory landscape the fly encounters at the quadrant border. As flies can move quite rapidly across the border region (~5–10 mm/s), they are expected to experience a significant rate of temperature change, with many interactions in the ~1 to 10 degrees per second range (Fig. 3g). In addition, considering the spatial separation of the antennae (and taking into account the steep gradient that our simulation predicts at the border region), the temperature at the aristae at any given point often differs by nearly 1 °C (Fig. 3g; note that, because of their tiny mass and the good thermal conductivity of chitin^22^, the aristae are expected to match the external temperature nearly instantaneously- see methods for a simple calculation illustrating this point).

Do flies use differential temperature readings from the antennae to chart the trajectory of U-turns away from heat? Many larger animals (e.g. owls^23^, rodents^24^, humans^25^, etc.) are well-known to use readings from bilaterally symmetrical sensory organs to orient in the environment, and both fly larvae and adult flies can use left-right asymmetries in the activity of olfactory neurons to orient towards an odor source^26–28^.

Whether similar mechanisms are used during thermotaxis is not known. Our simulation now makes it possible to address this question in highly controlled experiments in *Drosophila*, despite the small spatial scales involved. We set out to address the following questions: (1) can the thermosensory neurons of the antenna reliably detect the small temperature differences (0.1–1 °C) that would determine the direction of U-turns at hot/cool thermal boundaries? And is this information transmitted to downstream circuits in the brain? (2) Do asymmetries in the thermal stimuli at each antenna correlate with the left/right orientation of U-turns? And can experimental manipulation in the symmetry of TRN activation result in predictable left/right-turn bias?

To test the limits of sensitivity of the antennal TRNs, we utilized a preparation that allows one to challenge the antennae with highly controlled thermal stimuli^8^, designed to be as similar as possible to what the fly would encounter leading up to a U-turn at the border (see Supplementary Fig. 3 for a direct comparison). At the same time, we measured responses in the axon terminals of antennal hot TRNs using Calcium imaging and 2-photon microscopy (i.e. by targeted expression of the transgenic Calcium indicator GCaMP7f^29^, Fig. 3h–l).

Our observations suggest that, indeed, hot-activated TRNs of the arista can reliably respond to temperature stimuli as small as 0.1 °C (Fig. 3i, j). Moreover, imaging second-order thermosensory projection neurons (TPNs, by expressing GCaMP under the control of VT46265^11^) demonstrated that these cells are also reliably activated by such stimuli (Fig. 3i, j). This result shows that the response to small thermal stimuli is faithfully transmitted across the first central synapse to drive activity in central thermosensory circuits. Interestingly, in this range (Δ*T* < ~5 °C), the responses to thermal stimuli scaled with the magnitude of the stimulus for both TRNs and TPNs, and showed no sensitization following exposure to a larger stimulus (Fig. 3k, l; note that we will return to this point further below).

**Fig. 4:**
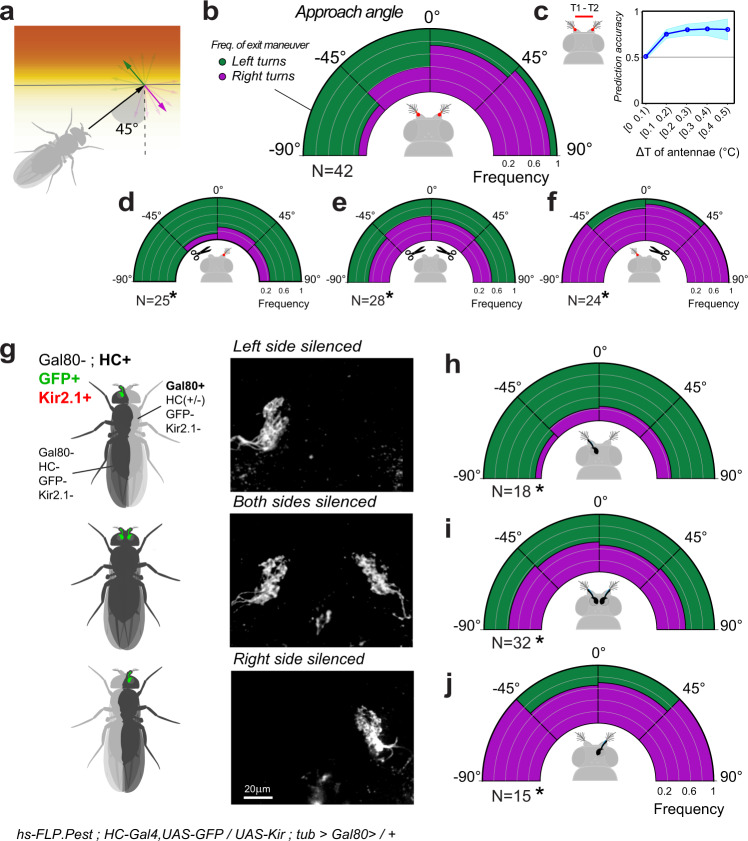
Differences in antennal input determine the direction of escape turns during thermotaxis. **a**–**c** For border interactions, the angle of heading is predictive of the angle of escape. **a** Schematic of the analysis. The heading angle is quantified in relation to the isothermal lines of the cool/hot boundary while the escape turn is categorized as “left” (green) or “right” (purple). **b** Distribution of left/right escape turns (binned in 45° intervals) as a function of initial heading angle. **c** Inter-antennal differences >0.1 °C are predictive of escape turn direction (prediction accuracy, bootstrap mean ± STD). **d**–**f** Surgical removal of the left (**d**) or right (**f**) antenna biases escape turn direction towards the side of the lesion (GLMM, Wald Test, *P*_left_ = 6.3e−5, *P*_right_ = 9.3e−4), while removal of both antennae (**e**) abolishes left/right bias (GLMM, Wald test, *P*_both_ = 2.1e−4). **g** Stochastic loss of the Gal4 inhibitor Gal80 produces flies in which either the left or right hot TRNs of the arista are genetically silenced and, at the same time, labeled by GFP expression (representative 2-photon stacks of TRN axon terminals are shown to the right, labels are the corresponding genotypes). **h**–**j** Stochastic silencing of either left or right hot TRNs (or both), produces a distribution of turning angles similar to that obtained from surgical ablation ((**i**) is the control genotype with bi-lateral silencing, see corresponding panel in (**g**)) (In all panels *N* denotes the number of animals, GLMM, Wald test, *P*_left_ = 9.0e**−**6, *P*_right_ = 2.1e−2; *P*_both_ = 2.5e−5). Note that mosaicism was determined by post-facto dissection and imaging.

Next, we tested the idea that flies may use the differential activation of antennal TRNs to chart an effective trajectory away from heat. In the simplest scenario, a fly approaching the hot/cool boundary head-on is expected to experience minimal differential activation of the antennae and is therefore likely to turn either left or right with equal probability. In contrast, a fly approaching the boundary from the left (i.e. forming an acute angle of approach with the boundary) would experience greater heat activation of the left antenna, and therefore may be more likely to turn right to escape the heat (Fig. 4a).

Our data suggest that, when considering the first turning maneuver a fly performs at the hot/cool boundary, this prediction indeed bears true: flies approaching the boundary head-on turn equally likely to the left or right; flies approaching from the left overwhelmingly turn right to escape the heat, and flies approaching from the right overwhelmingly turn left (Fig. 4b). Interestingly, an estimate of the temperature difference between antennae during those first turns (based on the simulation described above) suggests that temperature differentials as small as 0.1–0.2 °C are sufficient to predict turn direction (Fig. 4c).

To further test the idea that asymmetric activation of the antennae may determine turn direction, we ran our assays on flies from which either the right or left antenna had been surgically removed. Removal of the left antenna produced flies that, when approaching the hot/cool boundary, overwhelmingly turned left to escape -no matter the direction of approach. Conversely, removal of the right antenna produced flies that, when approaching the hot/cool boundary, overwhelmingly turned right (Fig. 4d, f). Importantly, left/right turn probability was not biased in these animals at constant 25 °C (Supplementary Fig. 4). Together, these results suggest that flies may interpret lack of input from the lesion side to indicate cooler conditions, and chart their escape turns towards the lesion side accordingly.

As an additional control, removal of both antennae completely abolished left/right turning bias -producing flies that turned either left or right with comparable frequency, no matter the angle of approach (Fig. 4e, but note that antenna-less flies perform U-turns much less frequently and—as a result—are more likely to invade the hot quadrants, see Fig. 2).

Next, we tested the extent to which the hot receptors of the arista may contribute to this left-right turn signal. We engineered flies in which transient activation of an FLP recombinase (i.e. under the control of a heat shock promoter^30^) leads to stochastic but permanent loss of the Gal4 inhibitor Gal80 in embryonic precursor cells that will eventually contribute to the adult body. In our set-up, clonal loss of Gal80 results both in genetic silencing (by expression of Kir2.1) and fluorescent labeling (by GFP) of cells that also express HC-Gal4 (i.e. hot TRNs of the arista). Because of early induction of the clones, the Gal80+ and Gal80- territories represent large areas of the body of mosaic animals, including flies in which TRNs are silenced in the left but not in the right antenna (Fig. 4g).

For this experiment, we ran ~200 putative mosaic flies in our behavioral assay and selected 33 asymmetrically silenced individuals for analysis by *post-facto* dissection and imaging (Fig. 4g, h, j). As a control, we used flies that did not express Gal80, and in which therefore hot TRNs of both antennae had been silenced (Fig. 4g, i).

The results of asymmetric silencing of hot TRNs (Fig. 4h, j) line up remarkably well with those obtained by surgical removal of either the left or right antenna. Silencing the hot TRNs of the left antenna produced flies that overwhelmingly turned left to escape the heat, while silencing the hot TRNs of the right antenna produced flies that escaped to the right (Fig. 4h, j**;** and see Supplementary Fig. 4 for additional controls). Together, these results demonstrate that creating an artificial asymmetry in the input from hot TRNs of the arista is sufficient to predictably bias turn direction at the hot/cool boundaries towards the silenced/ablated side.

Our results so far suggest that, upon encountering a hot/cool boundary, the fly uses differential information from the antennae to compute an efficient turn trajectory away from heat. This is reminiscent of the behavior of a “Braitenberg Vehicle”^1^, one of the simplest theoretical models of sensorimotor transformation, in which differential activity at two symmetrical detectors produces turns away from the source of a stimulus by controlling the speed of two symmetrical motors.

To what extent can the fly’s turning responses be explained by that of a simple “Braitenberg Vehicle” hard-wired for aversion? We reasoned that comparing the behavior of the fly to that of a simple vehicle model may reveal indications of plasticity and/or decision making that are not immediately obvious from analyzing fly behavior alone.

**Fig. 5:**
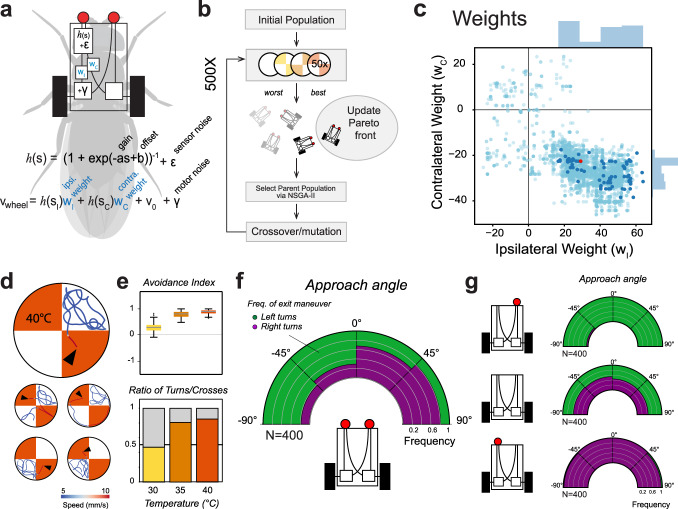
An evolved “Braitenberg vehicle” nearly reproduces fly thermotaxis. **a** An in silico “Braitenberg vehicle” model matching the dimensions of a fly, with key parameters used as substrate for evolution (*s* = sensory input, *v* velocity; parameters: *a* gain, *b* offset *ε, γ* noise (2 evolved parameters each, see methods), *w*_*i*_, *w*_*c*_ = weights of ipsi- and contralateral connections). **b** Schematic of the evolutionary process used to optimize the parameters. **c** Connectivity weights. Note that the best performing vehicles (dark blue dots) preserve both ipsi- and contralateral connectivity, and that ipsilateral weights are exclusively positive (excitatory) while contralateral weights are exclusively negative (inhibitory). **d**–**f** An evolved vehicle (red dot in (**c**)) nearly reproduces fly thermotactic behavior in a simulated arena. **d** Traces from a top-performing vehicle in the simulated arena (see Supplementary Fig. 5 and “methods” for details; arrowhead = start). **e**, **f** Vehicle performance in the simulated chamber (*N* = 400 simulations). **g** Vehicle responses are not robust to perturbation. “Ablation” of a single sensor produces vehicles that, entering the cool/hot boundary, invariably turn to the side of the lesion, irrespective of the direction of approach (mid-panel: as a control, removal of both sensors abolishes directional responses; *N* = 400 simulations each).

Towards this goal, we created an in silico vehicle scaled to the physical dimensions of the fly (e.g. same height and distance between the antennae, antennae and legs/motors etc.) and used an evolutionary algorithm to optimize eight free parameters (Fig. 5a–c, Supplementary Fig. 5a, b and see methods for details), selecting at each generation vehicles that, when tested in the simulated chamber, best matched the fly’s behavior in 4 key areas: probability of “spontaneous” turns at 25 °C; avoidance index for the 3 temperatures; fraction of U-turns/crosses at the border between 25° and 30°, 35° and 40 °C, and sensitivity to left/right temperature differences between the antennae (i.e. by matching the probability of a left/right turn given antennal temperature difference at turn start, see Fig. 4c). Amongst the free parameters that determine vehicle responses, two were used to define a simple sensory transformation, and two parameters specified the amplitude and time constant for each independent noise function (on sensor and motor output); we also added independent weights to the ipsilateral and contralateral sensor-to-motor connections (Fig. 5a, and see “methods” for details).

Our simulation assessed the performance of 42,042 vehicles over 500 generations of evolution. The final group of all-time best performers (the Pareto front), contained 559 vehicles, and 102 of those were chosen for further analysis based on good performance in all four criteria (the dark points in Fig. 5c and Supplementary Fig. 5b, d represent parameters from these vehicles). We finally chose the best performing vehicle to compare to fly behavior (but note that all 102 vehicles in the final group performed similarly, as shown by convergence of the four loss functions, Supplementary Fig. 5c, d).

Despite their inherent simplicity, after 500 generations our vehicles matched the performance of flies in the arena remarkably well (Fig. 5d, e and Movie S1). This included the distribution of U-turn left/right choices at the border (Fig. 5f), an objective not explicitly included in our selection process. We note that all top vehicles resulting from our evolutionary process retained both ipsi- and contralateral connectivity (i.e. the connectivity weights settled on non-zero values, see Fig. 5c). This suggests that coordination between left and right motors may be advantageous even in these very simple conditions.

Interestingly, while recapitulating well the fly’s turning responses at the border, the vehicle model failed to capture distinctive aspects of fly navigational behavior -ranging from the obvious to the more intriguing.

**Fig. 6:**
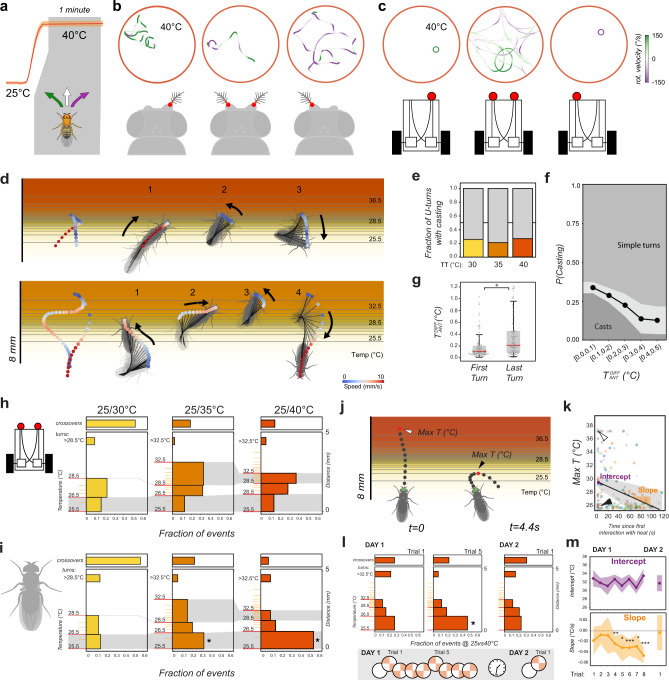
Comparison of thermotaxis in flies and vehicles reveals latent robustness and plasticity in fly behavior. **a**–**c** Under uniform heat conditions, fly behavior following antenna ablation is less stereotypical that vehicle behavior following sensor ablation. **a** Schematic of constant-heat experiment. **b**, **c** Representative tracks from (**b**) antenna ablated and control flies and (**c**) sensor ablated and control vehicles. Track color represents rotational speed (green = leftward rotation, purple = rightward rotation). Unlike flies, sensor-ablated vehicles rotate in place in uniform heat. **d**–**g** When navigating the cool/hot boundary, fly behavior is also less stereotypical than that of vehicles. Unlike vehicles, in a fraction of border interactions, flies perform “casting” (defined as at least two changes in direction in close succession) before escaping. **d** Two examples of casting behavior. **e** Fraction of escape turns that contain at least one casting event, plotted by experimental condition (*N*_30_ = 67/28, *N*_35_ = 114/30, *N*_40_ = 191/39 turns/animals). **f** Probability of casting is highest when the approach angle results in a small temperature difference between the antennae (*N* = 341 interactions from 95 flies; bins = 0.1 °C intervals, gray shading = ±STD, GLMM with Wald test, *P* = 1.5e−2, Coefficient = −3.12). **g** The last turn of a casting sequence is often characterized by a larger temperature difference between the antennae, compared with the first turn (box edges = first and third quartiles, solid line = median, whiskers = data range; *N*=92 casts from 55 animals, LMM, ANOVA, *P* = 2.2e−4). **h**–**m** Fly heat avoidance also displays hallmarks of rapid learning. **h**, **i** Compared to vehicles, flies display a disproportionate fraction of early turns (turns in the <26.5 °C region, lower gray shading) in the 25°/35 °C and 25°/40 °C experimental conditions. Histograms represent fraction of U-turns in different regions of the temperature gradients for (**h**) vehicles and (**i**) flies (left *y*-axis = temperature (°C), right *y* axis = distance (mm); gray shading = similar temperature range across conditions; crossover frequencies are shown at the top; asterisks in **i** = GLMM, Wald Test, *P*_35_ = 1.2e−7, *P*_40_ = 1.1e−26; **h**: *N*_30_ = 138/29, *N*_35_ = 131/25, *N*_40_ = 181/29 events/flies; **i**: *N* = 2493,3087, 3109 events/ 400 vehicle simulations each). **j**–**m** The dynamics of appearance of early turns suggests an underlying learning process. **j** Representative tracks showing a border crossing followed by an “early turn” (t = time from first border interaction; arrowheads = maximum temperature at the antennae, Max T, capped at 37 °C for crossings). **k** Border crossings and deep turns (leading to exposure to high heat) decrease during the course of an experiment in favor of early turns (LMM ANOVA, *P* = 1.3e−4; gray shading = 95% confidence interval; arrowheads in (**k**) correspond to events in (**j**); *N* = 28 flies). **l** When naïve flies are subjected to consecutive trials, early turns are significantly increased after five trials (plots as in (**h**, **i**); asterisk = GLMM, Wald test, *P* = 1.3e−2, *N* = 55 flies, *N*_events_: *N*_D1T1_ = 238, *N*_D1T5_ = 264, *N*_D2T1_ = 79). Early turn frequency returns to naïve levels after 24 h (right panel). **m** When considering the maximum temperature experienced at each border interaction, the initial exposure to heat remains constant across trials (intercept, top panel), but, after trial 4, flies rapidly resort to early turns as a strategy to escape heat (negative slope, bottom panel). This effect is reversed after 24 h of rest. Here, max temperature data were extracted and plotted as in (**j**, **k**) (points = coefficient from maximum likelihood estimation LMM, shading = 95% confidence interval from parametric bootstrap; asterisks = LMM ANOVA, *P*_4_ = 1.4e−3, *P*_5_ = 1.7e−2, *P*_6_ = 5.5e−5, *P*_7_ = 1.7e−2, *P*_8_ = 1.4e−9; in (**l**, **m**): *N*_day1_ = 55 flies, *N*_events_: *N*_1_ = 108, *N*_2_ = 268, *N*_3_ = 71, *N*_4_ = 338, *N*_5_ = 105, *N*_6_ = 280, *N*_7_ = 103, *N*_8_ = 268, *N*_day2_ = 13 flies, *N*_events_ = 71).

First, flies appeared better than vehicles at adapting to a change in sensory input state -i.e. as a result of removal of the left or right antenna/sensor. Like flies, sensor-ablated vehicles had a significant turning bias at cool/hot boundaries and overwhelmingly turned towards the side of the lesion when encountering heat (Fig. 5g). Yet, occasional entries in the hot region trapped the vehicles in a state of continuous spinning, a behavior not seen in flies (Movie S2). This effect was also observed in homogeneous heat conditions: unlike flies, sensor-ablated vehicles were unable to cope with uniform heat and remained trapped in continuous spins (Fig. 6a–c). Antenna-ablated flies initially turned towards the side of the lesion when exposed to homogeneous heating (Supplementary Fig. 6), but adapted their behavior to produce less stereotypical trajectories in constant heat (see Fig. 6b).

In addition to being less robust to new conditions, as may be expected, the vehicle’s maneuvers at the boundary appeared much more stereotyped than those of flies. As our algorithm does not directly select for stopping frequency, the vehicles did not perform the spontaneous stop-and-go that are typical of fly locomotor behavior. In particular, real flies in the arena slowed down considerably within the cool/hot boundaries, often coming nearly to a stop and performing side to side swings (reminiscent of “casting”^7,28,31^) before performing a sharp turn, and speeding up again to escape the heat (Fig. 6d).

This was not seen in vehicles—whose turns instead efficiently minimize the time spent within the hot area (i.e. by speeding up as the heat increases). We propose that this fly “casting” behavior may represent an information-gathering step that leads to a better informed turning decision. This idea is supported by two lines of evidence: (1) casting occurred in ~25% of U-turns (Fig. 6e), and was more likely in cases in which the initial border approach resulted in a small temperature differential between the antennae (perhaps reflecting initial uncertainty on escape direction; Fig. 6f). (2) A casting sequence may comprise multiple side-to-side swings (see Fig. 6d for an example), but the last turn of the sequence (the one leading to escape) generally started from a position characterized by a larger temperature differential between the antennae, compared to the first turn (Fig. 6g). Hence, the casting sequence often resulted in reduced uncertainty on the direction of escape.

Finally, we analyzed turning frequencies at the boundary as a function of temperature, and compared the results from flies and vehicles. The data revealed that flies resort remarkably rapidly to learned responses, even in this simple behavioral assay.

Our detailed simulation demonstrates that, at the height of the antennae, the cool/hot boundary is characterized by different temperature gradients in each of the 3 experimental conditions (25°/30 °C, 25°/35 °C, and 25/40 °C). For example, while the gradient in the 25.5–26.5 °C thermal range is very similar across the 3 conditions (lower gray shading in Fig. 6h, i), the 28.5–32.5 °C gradient is much steeper within the 25/40 °C than in the 25/35 °C boundary (upper gray shading in Fig. 6h, i).

As a result of a very similar initial gradient, the vehicle’s turning frequency is comparable across conditions in the 25.5–26.5 °C region (lower gray box in Fig. 6h). Beyond this point, vehicle turning frequency becomes higher in hotter regions (e.g. in the 25/35 °C condition) or in correspondence of the steepest gradient (25/40 °C, upper gray box in Fig. 6h).

Surprisingly, this was not the case for fly behavior. Flies’ turning frequencies appeared disproportionately high in the 25.5–26.5 °C initial region compared to vehicles’ turning frequencies, in particular in the 25/35 °C and 25/40 °C conditions. In fact, the fly’s propensity to perform “early turns” (turns in the initial part of the gradient) seemed to increase as a function of the test temperature (i.e. the frequency of early turns was higher in the 25/40 °C than in 25/35 °C experiments; lower gray box in Fig. 6i).

Moreover, one or more early turns often followed a “deep turn” (a turn that lead to exposure to higher temperatures) or a border crossing (Fig. 6j), and a quantification of all border interactions in the 25/40 °C condition demonstrates that deep turns and border crossings were significantly reduced over the course of a single experiment (i.e. within-trial, Fig. 6k). Hence, the frequency of early turns appears to increase following interactions with more intense heat (>35 °C).

We reasoned that this phenomenon could be explained either by sensitization (exposure to intense heat may boost the subsequent responses to mild heat) or as a result of more complex plasticity. For example, flies could rapidly learn to associate a temperature increase with the exposure to “dangerous” heat that follows it, and turn early within the gradient.

As noted before, we observed no sensitization in the neural responses to heat in TRNs and TPNs (using 2-photon microscopy, see Fig. 3), but this observation alone is not sufficient to exclude potentially sensitized responses further downstream.

To directly test the possibility that rapid learning may indeed explain the appearance of early turns, we designed the following experiment: rather than exposing flies to a full sequence of temperature choices as in previous runs, we exposed a cohort of “naïve” flies to 8 consecutive presentations of the choice between 25 and 40 °C (as usual in alternating spatial configuration, see schematics in Fig. 6l).

Indeed, naïve flies behave more like the hard-wired vehicles, and only after ~5 trials did we observe a significant increase in the fraction of early turns (Fig. 6l). This effect was reversible: allowing individual flies to recover for 24 h in fly food vials restored the behavior to its naïve state (flies seemed to have forgotten what they learned, Fig. 6l).

Intriguingly, the dynamic restructuring of turning behavior following heat exposure could again be observed within trial, but the effect of heat exposure appeared to depend on prior experience. When considering all border interactions as a function of temperature, both naïve and experienced flies responded similarly to each new presentation of the stimulus (i.e. performed deep turns and border crosses at the beginning of each new trial; Fig. 6m, intercept). Yet, unlike naïve flies, experienced flies rapidly adopted early turns as a strategy to minimize heat exposure (Fig. 6m, negative slopes; intercepts and slopes were calculated from data plotted as in 6k).

This complex dynamic, together with the fact that we observed no sensitization in the responses to heat in TRNs and TPNs (see above), lead us to conclude that learning, rather than neural sensitization, is likely to explain the difference in the frequency of early turns we observed between flies and the hard-wired vehicle. Hence, even in this simple assay, flies rapidly deploy learned programs to better adapt behavior to the specific features of the thermal environment.

## Discussion

Together, our results demonstrate how even a small poikilotherm such as the fruit fly possesses sophisticated mechanisms to navigate the thermal environment.

We show that, much like the more studied sensory systems of larger animals, fly thermosensation leverages input differences between symmetrical sensors (the antennal thermosensory neurons) to directly estimate the direction of change of a salient stimulus (increasing temperature). Rather than using this information to localize a prey^23^ or to move up an olfactory plume^26^, this differential reading is used to quickly draft an efficient escape trajectory away from dangerous thermal conditions.

This is different from what is known from *C. elegans*^32,33^, and even from fly larvae^7^, that instead perform thermotaxis by biasing stochastic turning decisions on the basis of temporal variations in thermosensory input. In these systems, turning maneuvers eliminate the need for detecting differences between the left and right sensors in the animal, instead converting an external gradient into a temporal pattern of input. In 1933, Wigglesworth and Gillett reported experiments on the blood-sucking *Rhodinus* from which a single antenna had been removed. Their observations (“As the insect probes, it turns sharply towards the side with the antenna”^34^) indeed suggest that the use of inter-antennal differences for heat navigation may be common in insects. We know little on how larger animals (e.g. those that lack antennae) may navigate a thermal landscape.

Beyond its relevance to thermotaxis, our work parallels efforts to produce quantitative models of sensory-motor transformations in *C. elegans*^35^, the fly larva^7,36–38^ and adult^38,39^, as well as in vertebrate model systems such as zebrafish^40,41^. It remains an open question to what extent, even in these relatively simple systems, sensory-motor transformations remain flexible, rather than being strictly determined by a combination of stimulus parameters.

A 3D simulation of the thermal landscape put us in the unique position to create a realistic virtual arena in which to evolve a “Braitenberg-inspired” vehicle model, directly testing the notion that fly heat avoidance may be controlled by a combination of simple hard-wired responses.

We note that the Braitenberg formulation is an intentionally simplistic one. Our sensor parameters are not designed to match what we know about thermosensory neurons, and our “circuit” formulation is rather simple compared to the complexity of the fly nervous system.

Nevertheless, after evolution of a number of key parameters, our Braitenberg-inspired vehicles performed remarkably well in the simulated arena, matching many of the characteristic features of fly thermotaxis. This suggests that the basic navigational responses to a hot front may be indeed controlled by a relatively simple set of transformations.

Yet, our vehicles appeared less robust to a sudden change in input (e.g. from sensor ablation) and fly-vehicle comparisons revealed features of fly thermotaxis that suggest an information gathering/decision-making process, as well as an unexpectedly rapid emergence of learned responses. Together, our results reveal additional layers of complexity within this seemingly simple insect behavior.

Animal navigation continues to be an essential source of inspiration for work involving autonomous robots and vehicles. Our approach shows that the reverse can also be true: comparing an intentionally bare vehicle-model to real animal responses can reveal aspects of natural behavior that defy reduction to a combination of fixed action patterns and hard-wired responses.

## Methods

### Fly strains

All fruit flies used in this study were bred and reared in a 12:12 day-night cycle, on a diet of standard cornmeal agar medium, at room temperature and controlled humidity. The following strains were used: Canton-special, *UAS-Kir2.1*, *UAS-DTI*, *HC-Gal4* (^8^), *HC-LexA* (^11^), *AC-Gal4* (^15^), *trpA1*^*1*^ (BDSC#26504, backcrossed 5 times), Df ^*trpa1*^ (Df(3 L)*ED4421;* BDSC#8066), Df ^*Gr28B*^ (*Df(2* *L)Exel7031*), *VT46265-Gal4*, *UAS-GFP*, *UAS-GCaMP7f*^29^, hs-FLPG5.PEST and *tubP*-*FRT* > *GAL80*-*FRT* > (^30^).

The Gr28b.d-LexA line used in this study labels the 3 hot arista TRNs which project to the hot glomerulus in the PAL and (as far as could be ascertained) no other neurons in the animal (see Fig. 1). The HC-Gal4 line used here strongly labels the 3 hot responding arista TRNs and no other neurons in the antenna, brain, or ventral nerve cord (VNC). Additional off-target expression includes ~1–2 neurons in leg tarsi and additional 1–2 putative chemosensory neurons innervating the sub-esophageal zone (SEZ; note that these projections do not respond to temperature in Ca2+ imaging experiments).

### Generation of *Gr28B* mutants and transgenes

*Gr28B* mutants were obtained by imprecise excision of a Minos insertion in the last exon of the *Gr28B* locus (*Mi{ET1}Gr28b*^*MB03888*^; BDSC#24190). Excision 8 caused a 207 bp deletion and the insertion of a 7-bp Minos footprint, producing a frame shift and premature stop in the coding sequence. As a control, we also generated a precise excision from the same insertion (*Gr28B* Exc66). In both cases, the full genomic Gr28B locus was sequenced to confirm the effect of excision. For rescue, we expressed a *Gr28B.d* cDNA in the *Gr28b* Exc8 mutant background using HC-LexA. To create 13LexAop-Gr28b.d transgene, we prepared a cDNA library from total mRNA extracted from *D. melanogaster* whole bodies using Superscript III reverse transcription (Life Technologies). We then amplified the full-length *Gr28B.d* cDNA with primers *Dmel* Gr28B.d FWD 5′- CAaaacATGTCATTTTACTTTTGCG-3′ and *Dmel* Gr28B.d REV 5′- AAACGATTAAAAATTTATTTCCAATC -3′ (Kozak sequence for *Drosophila* in lower case letters) also included in Supplementary table 1. Next, we cloned the PCR product into pCR™8/GW/TOPO® TA (ThermoFisher), confirmed its identity by sequencing, and then transferred it into a p13LexAop destination vector, created by ligating the Gateway® cassette from pMartini Gate C R2-R1 (Addgene plasmid #36436) cut with XhoI and XbaI into pJFRC19-13XLexAop2-IVS-myr::GFP (Addgene plasmid #2622).

### Two-choice behavioral assays for groups of flies

For two-choice assays for temperature preference^8^, we used 3 to 5 day old well-fed male flies grown under 12 h light: 12 h dark cycles, and tested them during the day. Flies were anaesthetized on ice and groups of 20 individuals were tested on an array of individually addressable Peltier tiles (1″ or 25.4 mm square) covered by thin, disposable, black masking tape (Thorlabs). Circular, 1.8″ (45.72 mm) spaces were laser-cut in an 1/8” (3.175 mm) acrylic sheet to form individual arenas centered over the intersection of 4 floor tiles (see Figs. 1b, 2a; note that serrated edges prevent flies from climbing on the arena wall). After loading flies, arenas were covered by glass (1.8 mm thick). Calibration was performed before each experiment using a FLIR infrared imaging system and a custom MATLAB script. In each trial, flies were given a choice between 25 °C (BT) and a test temperature (TT) in opposing quadrants for 3 min, the spatial configuration of BT/TT quadrants was then reversed for an additional 3 min. Every change in conditions was interleaved by a brief 30” step at 33 °C to ensure redistribution of the flies. For consistency, here we used a set sequence of test temperatures (BT/TT): 25°/25 °C, 25°/30 °C, 25°/15 °C, 25°/35 °C, 25°/20 °C, 25°/10 °C, 25°/40 °C.

To analyze temperature preference behavior, the position of each fly was recorded during the experiment under IR-light using a Chameleon3 USB camera (FLIR). The data was then processed in MATLAB to determine the position of each fly in each frame and to calculate an avoidance index (AI = [number of flies at BT−number of flies at TT]/total number of flies). For avoidance index measurements, the first 30” of each video recording (corresponding to disorderly redistribution of groups of flies) was not used for further analysis. AI values were evaluated using Kolmogorov–Smirnov tests (to test for normality), Spearman correlations (to check homogeneity of variances, threshold *P* = 0.05), and differences between experimental and control animals were tested by two-way ANOVAs. In addition, where appropriate, we used a standard two-tailed t-test (threshold *P* = 0.05) to determine if AIs were different from zero.

### Two-choice behavioral assays for single flies

Experimental conditions were essentially as described above, except flies were run individually instead of in groups. Unless otherwise stated, we used a set sequence of test temperatures (BT/TT): 25°/25 °C, 25°/35 °C, 25°/30 °C, 25°/40 °C. Tracking of single flies and all data analysis was done in Python. Basic image processing (edge detection and ellipse approximation of body) was done using openCV. To calculate an avoidance index for single flies, we tracked the centroid position of the fly for the duration of the trial and used the following equation AI = [time at BT−time at TT]/total time. To calculate the translation and rotational velocities at each time point, we determined the centroid position and angle of orientation of the fly. Velocity was projected along the body axis of the fly to obtain the velocities in the forward and sideways moving directions. Direction of movement was calculated using the heuristic that the vast majority of movement is in the forward direction, as done in^42^. In Fig. 2b, g, AI values and speeds were compared using 1 or 2-way ANOVA, as appropriate (threshold *P* = 0.05). We used a standard two-tailed t-test (threshold *P* = 0.05) to determine if AIs were different from zero. As shown in Fig. 6l, we also performed a repeated trial experiment, in which naïve flies were tested in a series of subsequent 25 vs 40 °C trials. Each trial lasted for 2 min. Subsequent trials were interleaved by a 30 s pause at constant temperature (33 °C). At each new trial, the spatial configuration of hot and cool tiles was flipped, as shown in the schematic in Fig. 6l. After a set of 8 trials (day 1), flies were individually collected and placed in food vials at 25 °C overnight. They were then tested again the next day (day 2).

Fly tracks were additionally segmented to identify maneuvers executed in the boundary region- between the hot and cool quadrants. The boundary region was defined as starting at the 25.5 °C isotherm and extending 5 mm into the hot quadrant (a position characterized by stable temperature in all three experimental conditions according to our simulation- see below). Maneuvers were classified as “U-turns” if the fly started on the 25 °C quadrant, invaded the border region, and eventually returned to the 25 °C quadrant. “Border crossings” were defined as events that terminated with exit on the hot side. Crossover-to-turn ratio was defined as # U-turns / (# U-turns + # border crosses). In order to compare the ratios of U-turns vs border crosses in control and experimental animals (while taking into account the potential impact of fly-to-fly idiosyncrasies) we used a generalized linear mixed model (GLMM) with fly ID as a random effect and Wald testing to determine significance (threshold *P* = 0.05; data shown in Fig. 2)(refs. ^43,44^). The generalized linear mixed model (GLMM) was:

1 $$Y = g(X\beta + Z\gamma ) + \varepsilon$$

where *X, β* are the predictor variables and their corresponding coefficients (fixed effects), *Z, γ* are the design matrix for random effects (i.e. fly ID) and corresponding coefficients, *g* is an inverse link function (i.e. logistic or linear), and *ε* is the residual.

### Surgical and genetic ablation of thermal input

Surgical removal of antennae (Figs. 2,4) was performed under CO_2_ anesthesia, on the first day after eclosion. Flies were allowed to recover for 3-4 days in food vials before testing. To generate mosaics (Fig. 4), *hs-FLP.PEST; HC-Gal4,UAS-GFP/UAS-Kir; tub* > *Gal80* > */+* first instar larvae were heat-shocked in a hot bath at 37 °C for 2 h. Freshly eclosed adult flies were collected and screened under a stereo microscope equipped with a fluorescent light source (Carl Zeiss, Dublin, CA, USA) to confirm GFP expression in the antennae (indicating successful loss of Gal80). After testing each individual fly for temperature preference, mosaicism in antennal GFP expression was confirmed by dissection and 2-photon imaging on a Prairie Ultima 2-photon microscope equipped with a Coherent Chameleon Ti:Sapphire laser, GaAsP PMT, and an Olympus 40 × 1.1 NA water immersion objective. For this experiment, we ran ~200 individual putative mosaic flies in our behavioral assay, and selected 33 asymmetrically silenced individuals for analysis by post-facto dissection and imaging.

### Simulation of thermal conditions

To better understand the thermal landscape experienced by the fly as it navigates within the arena, we developed a detailed simulation of the temperature conditions within the chamber, based on the thermal properties of air (and of the chamber’s materials) and well-established fluid dynamics and heat transfer principles^45^. Our simulation used the Boussinesq approximation for the Navier Stokes equations, which were nondimensionalized to the form:

2 $$\frac{{\partial {\boldsymbol{u}}}}{{\partial t}} + {\boldsymbol{u}} \cdot \nabla {\boldsymbol{u}} = - \nabla P + \sqrt {\frac{{Pr}}{{Ra}}} \nabla ^2{\boldsymbol{u}} + \widehat kT$$

3 $$\nabla \cdot {\boldsymbol{u}} = 0$$

4 $$\frac{{\partial T}}{{\partial t}} + {\boldsymbol{u}} \cdot \nabla T = \frac{1}{{\sqrt {PrRa} }}\nabla ^2T$$

where ***u***, *P*, and *T* are nondimensionalized velocity, pressure, and temperature. *Pr* and *Ra* are the well-known Prandtl and Rayleigh numbers, respectively, and $\widehat k$ is the vertical unit vector. No slip boundary conditions (i.e. $u = \overrightarrow 0$) were used for the walls of the cavity, and the sides were treated as insulating. We used Dirichlet boundary conditions for the Peltier plates lining the bottom of the chamber:

5 $$T|_{{\mathrm{cool}}\,{\mathrm{plate}}} = - 0.5,\,T|_{{\mathrm{hot}}\,{\mathrm{plate}}} = 0.5$$

and the Robin condition for the glass barrier that encloses the chamber at the top:

6 $$\frac{{\partial T}}{{\partial n}} = - \lambda \left( {T - T_0} \right)$$

where *T*_0_ =−0.5 and *λ* is a dimensionless coefficient referred to as the Biot number. The problem was solved numerically using a Chorin projection scheme written using the FEniCS finite element package in Python (here, we treated inertial terms explicitly and the viscous terms semi-implicitly using a Crank-Nicolson approach). The method was benchmarked as in Christon, Gresho, and Sutton^46^. As an additional test, we performed direct temperature measurements (using a thermocouple, Physitemp) above both the hot and cool side of the arena, and at both sides of the glass cover in all experimental conditions; the measured temperature matched simulations predictions within ±0.1 °C.

We note that, as predicted, our simulations show a small convective cell (~1 mm wide) centered at the interface between the cool and hot floor plates (Supplementary Fig. 2). This cell is expected to cause a very localized horizontal air flow at the interface boundary between tiles, with a local maximum flow velocity of ~1 mm/s over ~1 mm. The fly’s average walking speed in this region is significantly higher (~5 mm/s, max 10 mm/s) and therefore this air flow is unlikely to independently influence behavior in the boundary region (see also ref. ^47^). Given the small spatial scales involved the heat transfer process equilibrated very rapidly (~0.5 s), hence our further analysis considers steady-state temperature profiles.

Note that, for this work, we do not account for potential delays produced by the diffusion of hot/cold stimuli from the external environment to the temperature receptors within the arista. To understand the impact of this approximation, we determined the time for temperature to diffuse to the hot cells taking into consideration the known thermal properties of chitin^48^. Since the base of the arista is covered in an approximately 2 *μ*m layer of chitin^49^, the average diffusion time across this boundary layer is likely negligible:

7 $$d = \frac{{L^2}}{\alpha } = \frac{{\left( {2 \times 10^{ - 6}\,{\mathrm{m}}} \right)^2}}{{1 \times 10^{ - 7}\,{\mathrm{m}}^2/{\mathrm{s}}}} \approx 4 \times 10^{ - 5}{\mathrm{s}}$$

where α is the thermal diffusivity of chitin.

We also tested the potential impact of a different estimate of the height of the antennae on our two-dimensional temperature models (Fig. 3e). We find that key temperature gradient parameters are rather similar for a wide range of antennal heights (~500–900 μm, Supplementary Fig. 2). Here, we use an antenna height of 700 μm (consistent with our in-house measurements from pictures of standing flies).

### Functional calcium imaging of TRN and TPN responses to temperature

Calcium imaging of temperature stimuli was performed in a custom-built microfluidics chamber^8,11^. Sensory neuron or second-order projection neurons terminals were selectively imaged within the brain by transgenic targeting of G-CaMP7f expression (under the control of HC-Gal4 or VT46265-Gal4, respectively). In brief, dissections were performed in AHL (artificial hemolymph) such that a sufficient head cuticle was delicately removed to provide optical access to the brain while maintaining the connection to the antennae (the antennal nerve) intact. The preparation was then placed in a custom-built chamber, covered with a plastic coverslip, and placed on the two-photon microscope stage. Rapid temperature changes were achieved by controlling the temperature of the medium via a custom-built system consisting of a series of three-way valves (Lee Instruments, response time 2 ms) and Peltier elements independently controlling baseline, ‘hot’ and ‘cold’ flow, respectively. Temperature in the bath was recorded using a BAT-12 electronic thermometer (Physitemp, time constant 0.004 s, accuracy 0.01 °C). Stimulus parameters were chosen to capture as closely as possible the heating rate, magnitude, and duration a fly is exposed to in the time leading up to the first turn at the hot/cool border (see Supplementary Fig. 3b). To estimate heating rate and duration for moving flies, the starting point of each turn was defined as explained below and the start of the heating stimulus was set as the first time prior to turning with negligible heating (<0.001 °C/s). For the 2-photon experiment, average heating rate was calculated for the interval prior to the stimulus peak (with the same threshold for calculation of interval start). Images were acquired at 256 × 256 pixels resolution and 2X optical zoom at a rate of 4 Hz on a Prairie Ultima two-photon microscope with a Coherent Chameleon Ti:Sapphire laser, GaAsP PMT, and an Olympus 40 × 0.9NA water immersion objective. Acquired image sequences were processed in MATLAB. Base fluorescence was calculated using a 5 s window preceding each valve trigger. To determine if the smallest stimuli tested (0.1, 0.2, and 0.5 °C) elicited responses from TRN and TPN axon terminals different from zero peak fluorescence we used a standard two-tailed t-test (threshold *P* = 0.05).

### Fluorescent microscopy and image analysis

For imaging, we dissected 2–4 day old animals in phosphate-buffered saline (PBS). Confocal imaging of the head and antennae of HC-LexA flies expressing GFP (Fig. 1) was performed on a Zeiss LSM 510 META confocal microscope equipped with an argon 450–530 nm, helium-neon 543 nm and helium-neon 633 nm lasers and a Zeiss LCI Plan-Neofluar/0.8 DIC Imm Corr 25x objective at 512 × 512 pixel resolution. Images were processed in Fiji. Two-photon imaging of GFP expression in the brain and VNC was performed at 945 nm on a Prairie Ultima 2-photon microscope equipped with a Coherent Chameleon Ti:Sapphire laser, GaAsP PMT, and an Olympus 40 × 0.9NA water immersion objective at 512 × 512 pixel resolution and 1X optical zoom. Maximum projections were obtained from stacks using 1 μm steps. Images were processed using Fiji.

### AC neuron electrophysiology

Electrophysiological recordings from AC neurons (Supplementary Fig. 1) were performed using 2-photon guided patch-clamp electrophysiology^21^. In brief, we combined a UAS-GFP transgene (alone or in combination with UAS-Kir2.1) with AC-Gal4 and used GFP fluorescence as a guide to patch AC neurons under a 2-photon microscope.

### Analysis of turn direction

To analyze the relationship between incoming angle and turning direction within the hot/cool border regions (Fig. 4), we extracted the first turn performed within the boundary upon entry from the cool side. Here, “turns” were defined as segments containing a deviation from the fly’s trajectory resulting in a rotational velocity of at least 45°/s. A positive rotational velocity corresponds to a left turn, while a negative rotational velocity corresponds to a right turn. To define the incoming angle, the starting point of the turn was defined by stepping back along the fly track until the rotational velocity component changed sign or until there was no longer a monotonic decrease in velocity. The angle of the body axis at this location relative to the isothermal lines of the hot/cool boundary was considered as the incoming angle. Testing for changes in turning bias following ablation or silencing of antennae was performed using a GLMM with both approach angle and fly ID as random effects and Wald testing for significance (see above, threshold *P* = 0.05). For bilaterally ablated animals, this test was modified to test if removal of the antennae abolished predicted turning bias (i.e. based on the behavior of the non-ablated control).

To estimate accuracy in predicting turn direction based upon differential temperature readings at the antennae at turn initiation (Fig. 4c), we first estimated the temperature at each antenna at each turn’s starting point (as defined above). We then calculated an inter-antennal temperature difference (left-right). Here, a negative number indicated a cooler temperature at the left and therefore would predict a left turn, while a positive number would predict a right turn. If this prediction was met we assigned a value of 1 to the event, a zero otherwise. We then binned the data according to inter-antennal temperature difference using a bin size of 0.1 °C, and calculated a mean prediction accuracy by taking the mean of the string of 1 s and 0 s for each bin. Standard deviation was obtained by bootstrapping the data within each bin 1000 times.

In order to check for potential turning bias resulting from asymmetric ablation or silencing, we calculated left/right turning frequencies at constant 25 °C (note that “turns” are deviations in rotational velocity reaching at least 45° per second in magnitude). Frequencies were then calculated by taking the ratio of counts between left and right turns (Supplementary Fig. 4).

### “Braitenberg” vehicle simulation

We used an evolutionary algorithm to develop a “Braitenberg” vehicle-inspired model^1^ that could reproduce fly navigational responses in our arena. The vehicle model is intended to “navigate” a realistic virtual arena, with a thermal landscape derived from our simulation (described above), and was therefore developed to be consistent with the physical dimensions of a real fly.

We considered a 2-wheeled description of movement dynamics of the form:

8 $$\begin{array}{*{20}{r}} \hfill {\left[ {\begin{array}{*{20}{c}} {x^\prime } \\ {y^\prime } \\ {\theta ^\prime } \end{array}} \right] = \left[ {\begin{array}{*{20}{c}} {\frac{1}{2}(v_L + v_R){\mathrm{cos}}(\theta )} \\ {\frac{1}{2}(v_L + v_R){\mathrm{sin}}(\theta )} \\ {\frac{1}{d}(v_R - v_L)} \end{array}} \right]}\end{array}$$

where *x* and *y* are the positions of the centroid and θ is the orientation. The wheels are independently driven, but fixed in their orientation relative to the body axis of the vehicle. Here, *v*_*L*_ and *v*_*R*_ are the velocities of the left and right motor, respectively, while *d* is the distance between the two wheels (set to 750 μm, reasonably close to a fly’s width).

The speed of the motors is controlled by two symmetrical sensors, with positions:

9 $$\left( {x_{LA},y_{LA}} \right) = \left( {x + \frac{{{\rm{BL}}}}{2}{\mathrm{cos}}\left( \theta \right) - \frac{{{\rm{AD}}}}{2}{\mathrm{sin}}\left( {\theta} \right),y + \frac{{{\rm{BL}}}}{2}{\mathrm{sin}}\left( \theta \right) + \frac{{{\rm{AD}}}}{2}{\mathrm{cos}}\left( {\theta} \right)} \right)$$

10 $$\left( {x_{RA},y_{RA}} \right) = \left( {x + \frac{{{\rm{BL}}}}{2}{\mathrm{cos}}\left( \theta \right) + \frac{{{\rm{AD}}}}{2}{\mathrm{sin}}\left( {\theta} \right),y + \frac{{{\rm{BL}}}}{2}{\mathrm{sin}}\left( \theta \right) - \frac{{{\rm{AD}}}}{2}{\mathrm{cos}}\left( {\theta} \right)} \right)$$

where BL (body length) = 3 mm and AD (antennal distance) = 300 μm (so that the sensors are located 300 µm apart at the front of the vehicle, and the motors 1.5 mm along the body axis separated by *d*). The base velocity *v*_0_ was set to 5 mm/s. BL, AD, sensor position and *v*_0_ were chosen to be reasonably close to a fly’s.

The sensory input used in the model derives from our temperature simulation of the behavioral arena (described above) and is additively modulated with time-correlated noise generated by an Ornstein–Uhlenbeck model:

11 $$\tau _s{\rm{d}}\varepsilon = - \varepsilon {\rm{d}}t + \sigma _s{\rm{d}}W$$

where *τ* is a time constant for the process, σ_*s*_ is a parameter that defines amplitude of the noise, *dW* denotes the Wiener process. Temporal integration of the differential equation was performed using the Euler–Maruyama method. Effective sensory input is given as

12 $$s_L = s_{L0} + \varepsilon _L$$

13 $$s_R = s_{R0} + \varepsilon _R$$

where *s*_L0_, *s*_R0_ are the temperatures at the left and right sensor, respectively, and *ε*_*L*_, *ε*_*R*_ are the noise values at each sensor (note that noise is not correlated between L and R sensors).

Another Ornstein-Uhlenbeck process was used to describe motor noise γ

14 $$\tau _m{\rm{d}}\gamma = - \gamma {\rm{d}}t + \sigma _m{\rm{d}}W$$

where τ_*m*_ is a time constant for the motor noise process, and σ_*m*_ is a parameter that defines the amplitude of the noise. The velocities of the wheels are linear combinations of sensory input (*s*_*L*_*, s*_*R*_) processed through a logistic nonlinearity at the two symmetric sensors and additively combined with the motor noise:

15 $$v_L = f_L\left( {s_L,s_R} \right) = h\left( {s_L} \right)w_{L,L} + h\left( {s_R} \right)w_{L,R} + v_0 + \gamma$$

16 $$v_R = f_R\left( {s_L,s_R} \right) = h\left( {s_L} \right)w_{R,L} + h\left( {s_R} \right)w_{R,R} + v_0 - \gamma$$

Note that the effect of the motor noise is anti-correlated for L and R wheel speeds in order to impact turning bias without altering overall speed.

The weights:

17 $$W = \left[ {\begin{array}{*{20}{c}} {w_{L,L}} & {w_{L,R}} \\ {w_{R,L}} & {w_{R,R}} \end{array}} \right]$$

relay the transformation of sensory input into left and right wheel speeds (note that the vehicle wiring is symmetric, as W is symmetric and $w_{L,L} = w_{R,R}$). *h* is a transformation of the sensory input defined by the logistic function $h\left( s \right) = \frac{1}{{1 + \exp \left( { - as + b} \right)}}$. To prevent the vehicle from getting stuck on the external border wall of the simulated chamber we specified that, upon collision with the wall, the orientation of the vehicle would be reflected about the wall normal vector and reversed as in a simple ballistic collision.

### Evolutionary optimization of vehicles

Multi-objective optimization of the vehicles was performed via an evolutionary strategy using the Non-dominated Sorting Genetic Algorithm II (NSGA-II) method available in DEAP Python package^50,51^. We optimized the vehicles to best match four objectives (*i.e*. by minimizing the Euclidean distance between the performance of the vehicle and fly for each objective): (1) the avoidance index and (2) turn-cross ratio at each of the three test temperatures, (3) the probability of a left/right turn given antennal temperature difference at turn start, and (4) the “spontaneous” rate of turns per distance walked. Optimization was performed over an eight-dimensional space, ${\boldsymbol{z}} = \{ w_I,w_C,\tau _s,\sigma _s,a,b,\tau _m,\sigma _m\}$ containing the ipsilateral and contralateral weights ($w_{L,L},w_{R,R} = w_I$ and $w_{L,R},w_{R,L} = w_C$), sensory noise parameters ($\tau _s,\sigma _s$), nonlinearity parameters (*a,b*), and motor noise parameters ($\tau _m,\sigma _m$).

At each new generation, the algorithm either “crossed” or “mutated” (each with probability 0.5) individuals from the previous round. The best performing individuals based on 200 trial simulations (comprised of 50 simulations of 25°/25 °C, 25°/30 °C, 25°/35 °C, and 25°/40 °C, each) were then selected using the NSGA-II method, which in addition includes an explicit diversity preserving mechanism to prevent convergence to local minima. The Pareto front for the four-dimensional objective space was updated following each new generation. After 500 generations of evolution (each with 112 individuals) we observed strong convergence of the error in each performance criterion among members of the Pareto front (Supplementary Fig. 5c, here, we consider the error in each objective of a particular vehicle, $e_i$, normalized by the median error in that objective of all final Pareto front members, $\gamma _i$, $Error_i = \frac{{e_i}}{{\gamma _i}}$). Following evolution, we compared the 102 all-time best performing vehicles in all four criteria (members of the final Pareto front that had no error greater that 4X the median error for any objective). The top-performing vehicle, defined as the vehicle with highest minimum rank across the four objectives (ranking according to magnitude of error in the objective), was used for comparisons with flies (see Fig. 6; *w*_I_ [mm/s] = 29.1, *w*_C_ [mm/s] = −22.5, $\tau$_*S*_ [s] = 0.75, σ_S_ = 0.0067, a [1/°C] = 0.5, *b* = 3.9, $\tau$_*M*_ [s] = 0.65, σ_M_ = 0.39). However, we note that all of the 102 best-performing vehicles performed similarly well (Fig. 5 and Supplementary Fig. 5).

### Analysis of casting

To quantify casting we segmented maneuvers executed in the boundary region as described above. A “cast” was defined as an event containing at least one left and one right turn (as defined above) in close succession. We then quantified the fraction of border interactions that contained at least one cast for each test temperature (Fig. 6e). To test the relationship between initial inter-antennal temperature difference (at start of first turn) and the probability of performing multiple turns in the boundary region (Fig. 6f), we used a GLMM with fly ID as a random effect and Wald testing to determine significance (threshold *P* = 0.05). For plotting in Fig. 6f, cast probability was calculated within each bin as *P*(casting) = casts/ (simple turns + casts). To estimate the potential change in inter-antennal temperature difference between the first and last turn within a cast (Fig. 6g), Δ*T* at the start of first and last turns was compared using a Linear Mixed Model (LMM) with fly ID as a random effect and ANOVA for significance (threshold *P* = 0.05).

### Uniform heat experiments

For experiments testing the response of flies to uniform heat or heating (Fig. 6a–c and Supplementary Fig. 6), the arena was heated uniformly from 25 °C to 40 °C and fly movement was recorded during a defined heating window (10 s, from ~28 °C to ~38 °C) or at stable temperature (40 °C). To establish the direction of the first turn induced by heating, we limited our analysis to flies that happened to be stationary at the beginning of the heating period. Vehicle simulations in constant heat were designed to match the conditions of fly experiments.

### Analysis of maximum temperature experienced

Boundary region tracks were analyzed over the course of each 25 vs 40 °C individual fly trial and the position of the head at maximum distance into the hot region was recorded (for schematic see Fig. 6j). The plot in Fig. 6k was constructed using the maximum temperatures reached during each interaction with the hot boundary (the first interaction with the border was considered time zero). To test for significance of the decreasing trend (while taking into account the potential impact of fly-to-fly idiosyncrasies) we used a linear mixed model (LMM) with fly ID as a random effect and ANOVA for testing (threshold *P* = 0.05). In Fig. 6h, i, l, we binned boundary foray depths using the positions of select isotherms. The differences in turning frequency in the 25.5–26.5 °C temperature bin between vehicles and flies, as well as between trials in the repeated trials experiment were tested using a generalized linear mixed model with fly ID as a random effect and Wald testing to determine significance (threshold *P* = 0.05).

### Reporting summary

Further information on research design is available in the Nature Research Reporting Summary linked to this article.

## Supplementary information

Supplementary Information

Description of Additional Supplementary Files

Supplementary Movie 1

Supplementary Movie 2

Reporting Summary

## Data Availability

All data are available in the main test or the supplementary materials.  Source data are provided with this paper.

## Source data

Source Data

## Code availability

Custom computer code is available from the corresponding authors on reasonable request.
