# Supplementary material for: Robustness and plasticity in Drosophila heat avoidance
Source: Nat Commun. 2021 Apr 6;12:2044. doi: 10.1038/s41467-021-22322-w (PMC8024383; doi:10.1038/s41467-021-22322-w)
Supplement: Supplementary file 1 — Supplementary Information [file 41467_2021_22322_MOESM1_ESM.pdf]

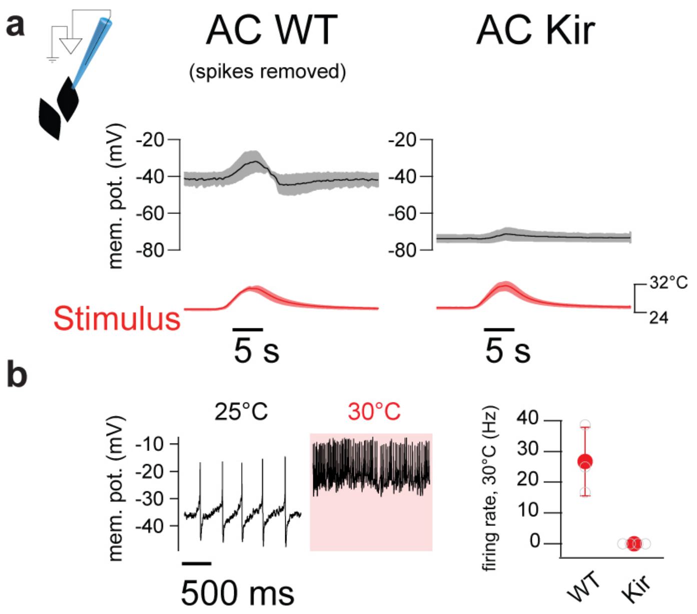

Supplementary Figure 1 | Kir2.1 effectively silences AC neurons.

Whole-cell current clamp recordings show genetic silencing of Anterior Cells (AC) by Kir2.1 expression is effective. **a.** Filtered membrane potential traces (spikes removed) from wild type AC neurons and from AC expressing Kir2.1. Kir2.1 expression hyperpolarizes AC cells and prevents the response to a ~5°C hot stimulus (red trace below; traces are average ± SEM from 3 WT cells or 4 Kir cells /5 repeats per cell).

b. Representative raw traces (left) recorded from a wild-type AC neuron showing increased firing in response to a temperature stimulus, and (right) quantification of firing rates from unfiltered recordings from wild type and Kir2.1 expressing AC neurons (plot is average ± STD; 3 WT cells and 4 Kir cells /5 repeats per cell).

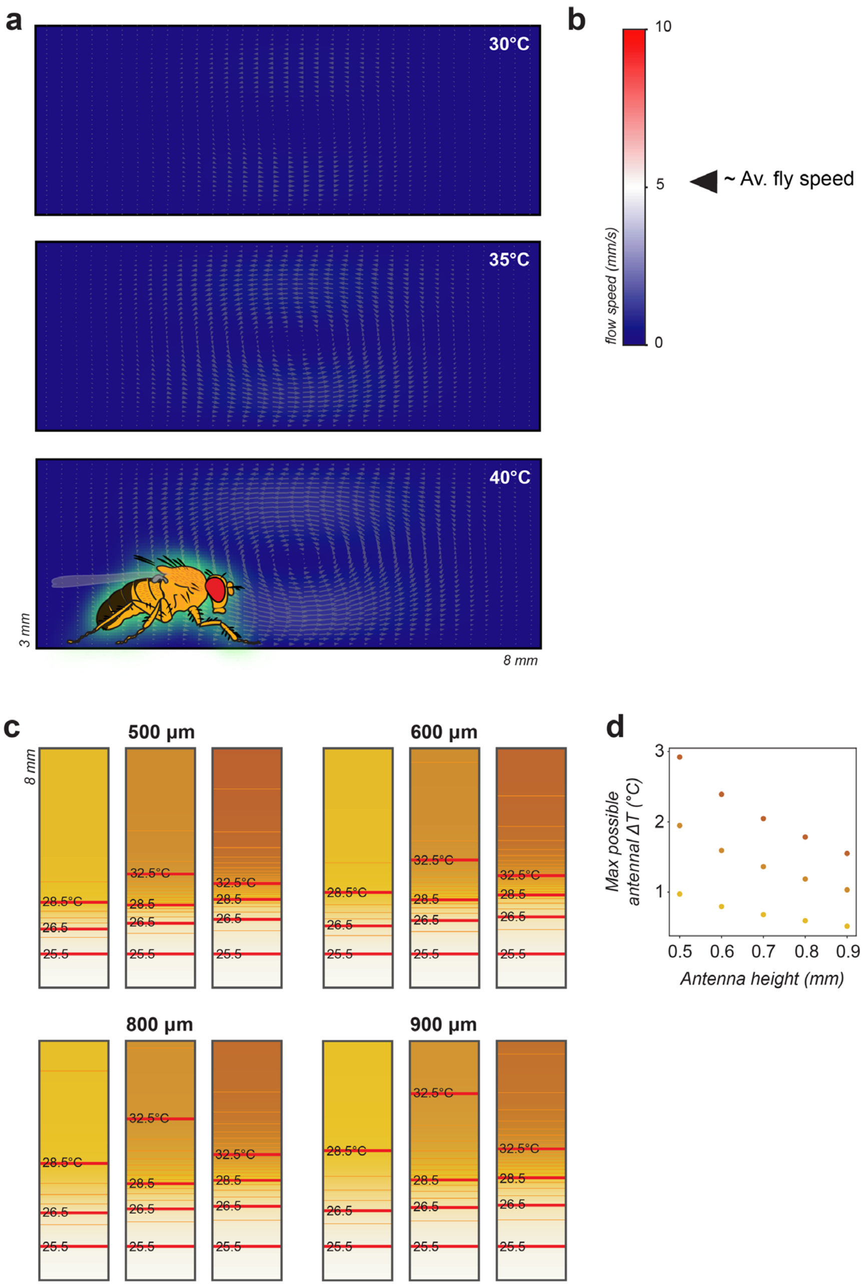

Supplementary Figure 2 | 3D simulation of the thermal environment: impact of convection and sensitivity of 2D models to height.

a. A simulation of the thermal conditions inside the arena predicts a small convective cell centered at the interface between the cool and hot floor plates. This cell is expected to cause a localized horizontal air flow (represented by arrows) above the interface boundary between cool and hot tiles. Flow velocity is represented by color (see scale bar in **b**; note that the arrow length in **a** is also proportional to flow velocity). The fly's average walking speed in this region is ~5 mm/sec (max ~10 mm/sec), and is also indicated on the scale bar in **b** for comparison. Because of the limited localization and speed of this air flow, we considered it unlikely to independently influence behavior in the boundary region. **c.** Predicted thermal gradient at different heights relative to the chamber floor (e.g. for different estimates of the height of the antennae). **d**. Maximum possible temperature differential between the antennae given the parameters in **c** (colors represents experimental conditions, yellow =25°C vs 30°C, orange =25°C vs 35°C, brown =25°C vs 40°C).

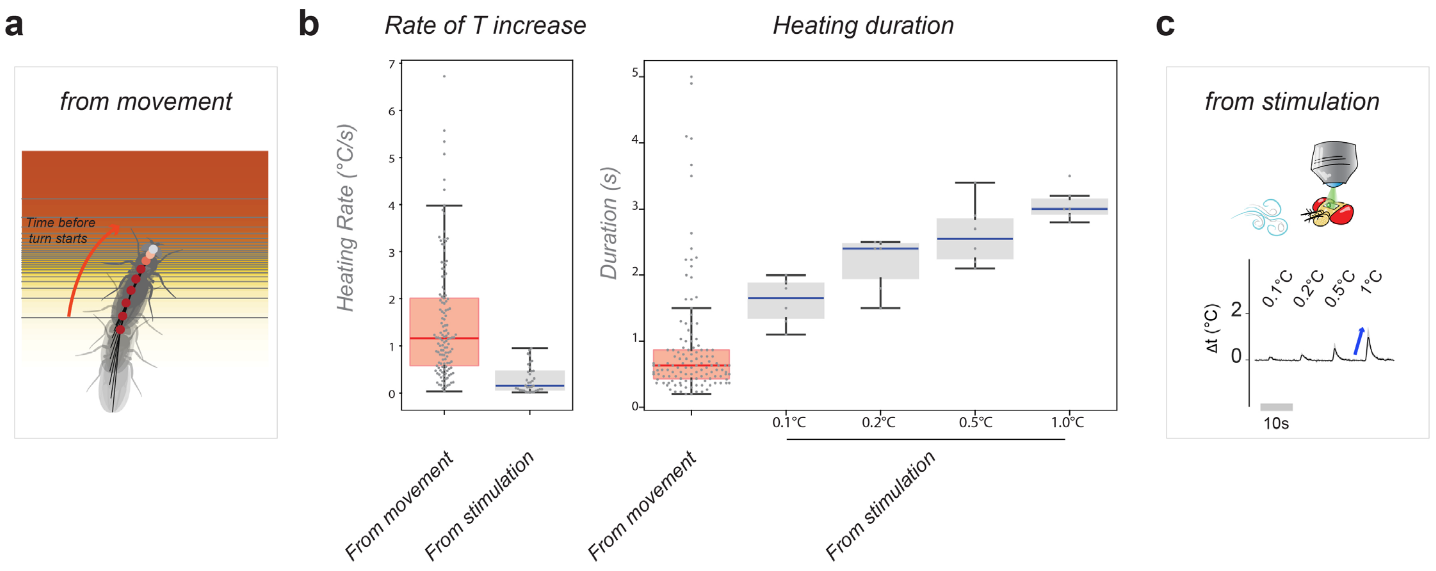

Supplementary Figure 3 | Comparison of stimulus parameters in 2-choice experiment and 2-photon imaging.

a. Diagram of fly moving through the thermal gradient -magnitude and duration of heating were estimated from the point of entry into the gradient to the starting point of a turn (see methods for details). **b.** Heating rate and duration experienced by flies leading up to a turn (red boxes, $N_{movement}$= 129 from 28 flies) shown for comparison next to the stimuli used during 2-photon microscopy experiments (grey boxes, N_{stim}=36 from 6 animals). **c.** Representation of a typical 2-photon microscopy experiment -rate and duration of heating were measured from baseline to peak temperature (blue arrow). In all boxplots, the edges of the boxes are the first and third quartiles, a solid line marks the median, and whiskers delimit the data range.

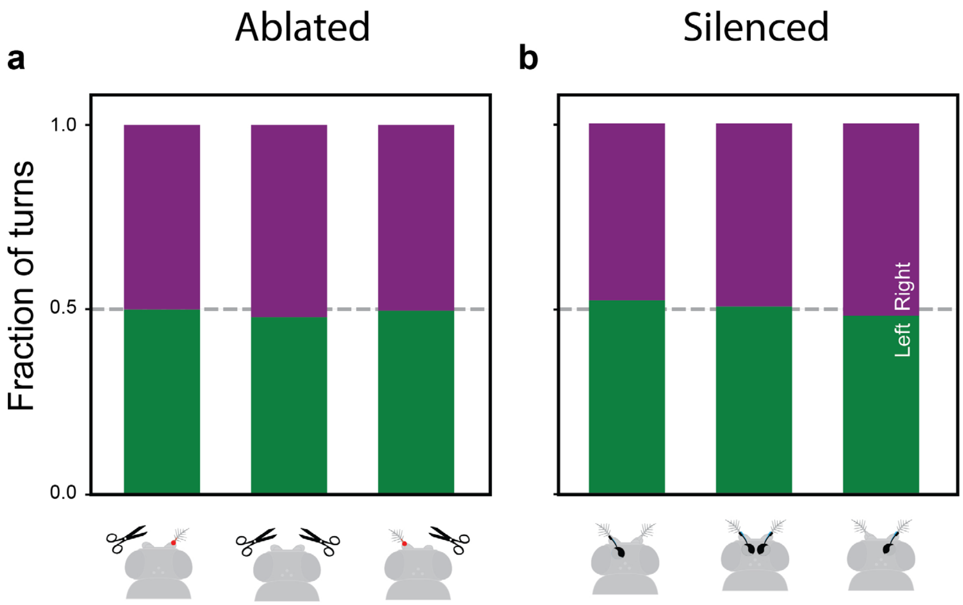

Supplementary Figure 4 | Ablation of the antennae does not bias turning direction at 25°C.

a. Physical ablation of the right antenna, both antennae, or of the left antenna does not bias turning direction at 25°C ($N_{Lablated}$= 32, $N_{L+Rablated}$= 38, $N_{Rablated}$= 27). **b.** Similar results are obtained for genetic silencing of hot receptor neurons (by HC>Kir2.1; see Figure 3, $N_{Lsilenced}$= 33, $N_{L+Rsilenced}$= 36, $N_{Rsilenced}$= 23). Plots show ratio of left/right turns at 25°C.

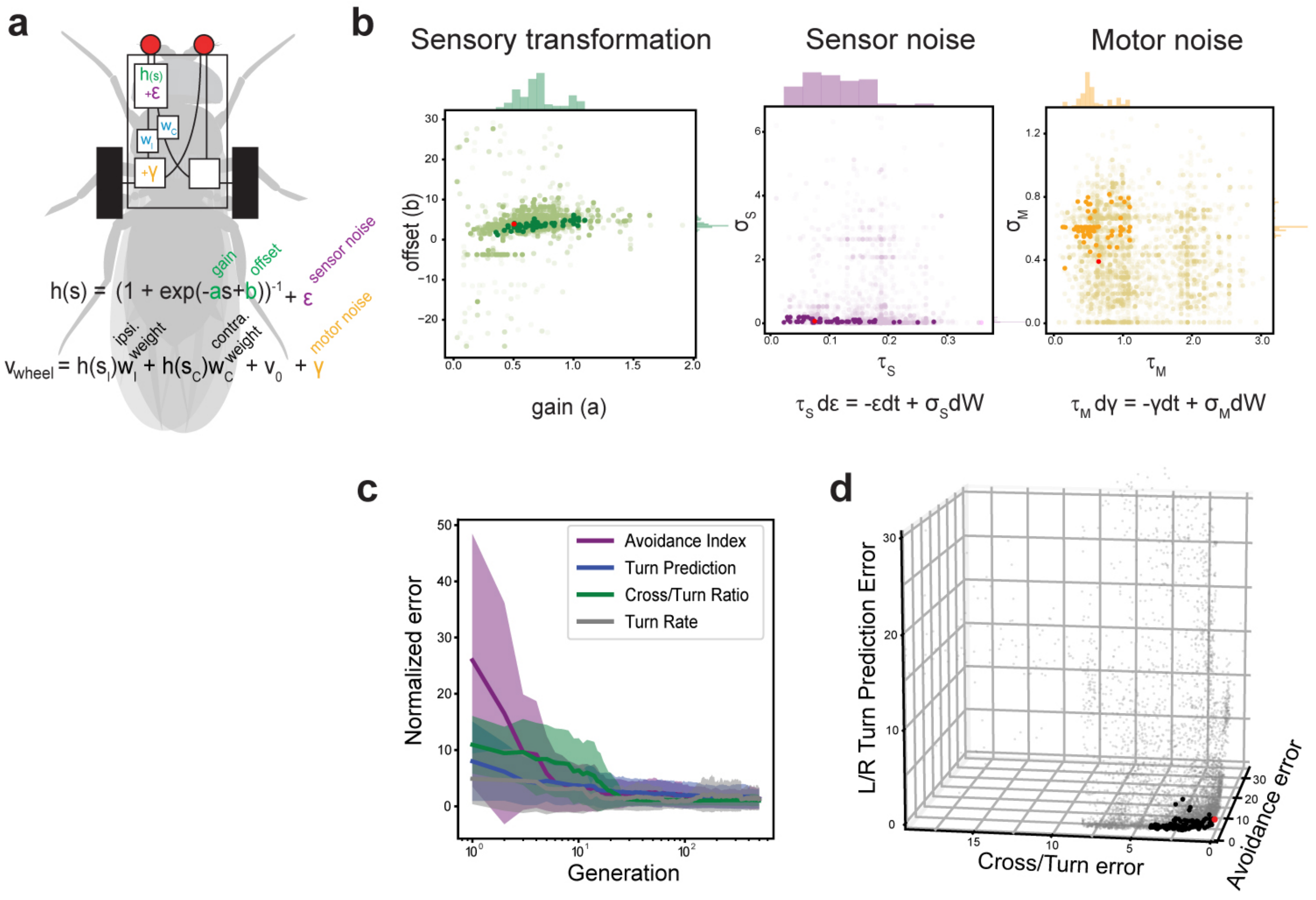

Supplementary Figure 5 | Parameter space explored during vehicle evolution.

a. Schematic of the vehicle model, indicating some of the functions and parameters used for evolution. **b.** Parameter space explored by six of the eight variables allowed to change during 500 generations of evolution (see main figure for ipsilateral and contralateral weight). Light-color points represent all vehicles tested during evolutionary optimization (N=42042 unique vehicles), dark-color points are from all-time best performers in all four objective functions (see methods for details; N=102), red point represents the best performing vehicle chosen for comparison with flies. Left panel (sensory transformation), *a,b* = evolved variables. Center panel, sensor noise ε, σ_S and τ_S = evolved variables. Right panel, motor noise γ, σ_M and τ_M = evolved variables. **c**. The error in each of the four objective functions converges following evolution (median ± median absolute deviation; error values are from each generation's Pareto front vehicles, the error of each vehicle is normalized by the median error of the final Pareto front vehicles) **d.** 3D scatter plot showing the error space for 3 key objectives of all vehicles tested (grey), the all-time best performing vehicles after 500 generations (black), and the top performing vehicle (red). X-axis = Crossover/U-turn ratio error, Y-axis = avoidance index error, Z-axis = Left/Right turn predictability error.

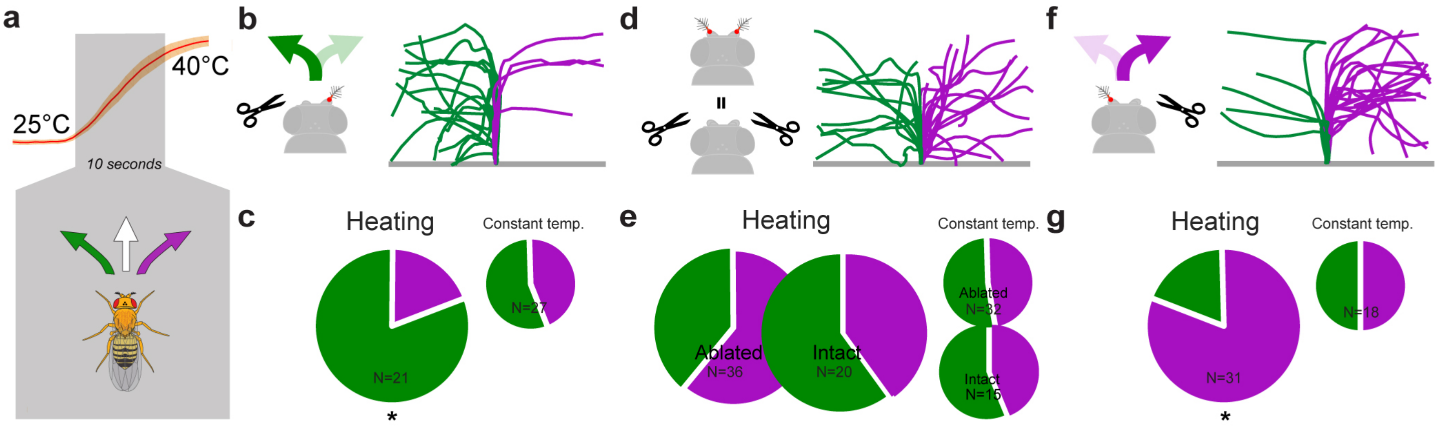

Supplementary Figure 6 | The behavior of antenna-ablated flies during uniform heating.

a. Experiment schematic. **b-g**. Analysis of the direction of the first turn performed by intact and antenna-ablated flies under uniform heating. **b,c.** Left antenna ablated. **d,e.** Both antennae ablated. **f,g.** Right antenna ablated. **b,d,f.** Fly tracks that include the first turn a fly performed upon heating (green=left turn, purple=right turn). **c,e,g.** Quantification of left/right turning frequencies in antenna-ablated flies (left/right turning frequency at constant 25°C is shown as a control; N=number of flies tested; asterisks denote a difference from the expected control distribution of 1:1, Chi-Squared test, P_{left} = 4.6e-3, P_{right} = 1.6e-4).

Primer name	Sequence
*Dmel*Gr28B.d FWD	5’- CAaaacATGTCATTTTACTTTTGCG-3’
*Dmel*Gr28B.d REV	5’- AAACGATTAAAAATTTATTTCCAATC -3’

Supplementary Table 1 | PCR primers
