# Supplementary material for: Robustness and plasticity in Drosophila heat avoidance
Source: Nat Commun. 2021 Apr 6;12:2044. doi: 10.1038/s41467-021-22322-w (PMC8024383; doi:10.1038/s41467-021-22322-w)
Supplement: Supplementary file 2 — Description of Additional Supplementary Files [file 41467_2021_22322_MOESM2_ESM.pdf]

Description of Additional Supplementary Files

File Name: Supplementary Movie 1
Description: **Drosophila flies and a model vehicle perform heat avoidance**
(Left) A single fruit fly avoids quadrants of the arena whose floor tiles are set to a hot temperature of 40°C (instead of the preferred 25°C). At the border between cool and hot quadrants, the fly performs characteristic U-turns. (Right) A two-sensor/two-motors “Braitenberg vehicle” model scaled to the real dimensions of the fly performs the same heat avoidance task, in a simulated chamber that reproduces the temperature profiles the real fly is expected to encounter (see text and methods for details).

File Name: Supplementary Movie 2
Description: **The model vehicles are unable to adapt to asymmetry in sensory input**
A thermosensory “Braitenberg vehicle” evolved to perform thermotaxis on the basis of a two-sensor/two-motors design is modified by removal of one sensor. As a result of asymmetric input, the vehicle displays a strong turning bias when encountering a hot boundary (towards the side of the missing sensor, see figures for details). Moreover, an occasional entry into the hot region traps the vehicle in a state of continuous spinning.
