# Supplementary material for: Robustness and plasticity in Drosophila heat avoidance
Source: Nat Commun. 2021 Apr 6;12:2044. doi: 10.1038/s41467-021-22322-w (PMC8024383; doi:10.1038/s41467-021-22322-w)
Supplement: Supplementary file 5 — Reporting Summary [file 41467_2021_22322_MOESM5_ESM.pdf]

nature research

Corresponding author(s): Marco Gallio

Last updated by author(s): Feb 8, 2021

Reporting Summary

Nature Research wishes to improve the reproducibility of the work that we publish. This form provides structure for consistency and transparency in reporting. For further information on Nature Research policies, see Authors & Referees and the Editorial Policy Checklist .

Statistics

For all statistical analyses, confirm that the following items are present in the figure legend, table legend, main text, or Methods section.

n/a	Confirmed	
☐	☒	The exact sample size (n) for each experimental group/condition, given as a discrete number and unit of measurement
☐	☒	A statement on whether measurements were taken from distinct samples or whether the same sample was measured repeatedly
☐	☒	The statistical test(s) used AND whether they are one- or two-sided<br>*Only common tests should be described solely by name; describe more complex techniques in the Methods section.*
☐	☒	A description of all covariates tested
☐	☒	A description of any assumptions or corrections, such as tests of normality and adjustment for multiple comparisons
☐	☒	A full description of the statistical parameters including central tendency (e.g. means) or other basic estimates (e.g. regression coefficient) AND variation (e.g. standard deviation) or associated estimates of uncertainty (e.g. confidence intervals)
☐	☒	For null hypothesis testing, the test statistic (e.g. F, t, r) with confidence intervals, effect sizes, degrees of freedom and P value noted<br>*Give P values as exact values whenever suitable.*
☒	☐	For Bayesian analysis, information on the choice of priors and Markov chain Monte Carlo settings
☐	☒	For hierarchical and complex designs, identification of the appropriate level for tests and full reporting of outcomes
☒	☐	Estimates of effect sizes (e.g. Cohen's d, Pearson's r), indicating how they were calculated

Our web collection on statistics for biologists contains articles on many of the points above.

Software and code

Policy information about availability of computer code

Data collection	Collection of behavioral data was done using a custom MATLAB script. Calcium imaging recordings were done using Prairie View software PVScan version="5.2.64.400"). Electro-physiological recordings were performed using pClamp. Microscopy panels were processed in Fiji.
Data analysis	Analysis of group behavior was done using a custom MATLAB script. Individual fly behavior analysis was done using custom Python scripts. Analysis of calcium imaging was done using custom MATLAB scripts, analysis of electro-physiology was done in Axograph.

For manuscripts utilizing custom algorithms or software that are central to the research but not yet described in published literature, software must be made available to editors/reviewers. We strongly encourage code deposition in a community repository (e.g. GitHub). See the Nature Research guidelines for submitting code & software for further information.

Data

Policy information about availability of data

All manuscripts must include a data availability statement. This statement should provide the following information, where applicable:

- Accession codes, unique identifiers, or web links for publicly available datasets
- A list of figures that have associated raw data
- A description of any restrictions on data availability

The data generated during the current study are available as a source data file and from the corresponding author on reasonable request. Fly strains will be freely distributed without restrictionas is customary in the field.

Field-specific reporting

Please select the one below that is the best fit for your research. If you are not sure, read the appropriate sections before making your selection.

Life sciences study design

All studies must disclose on these points even when the disclosure is negative.

Sample size	Sample sizes were not predetermined based on statistical methods, but were chosen according to standards in the field (eg, Frank et al, Nature 2015; Alpert et al, Current Biology 2020). All papers that report data on fly behavior, electro-physiology or imaging use similar sample sizes. All group experiments included four or more groups, all individual behavioral experiments included >10 replicates, and all calcium imaging and electro-physiology experiments used at least 5 biological replicates, which generated sufficient data to capture the phenomena of interest.
Data exclusions	No data was excluded from analysis.
Replication	Reported results were consistently replicated across multiple experiments, with all replicates generating similar results.
Randomization	Flies of the same genotype were randomly allocated to experimental groups.
Blinding	Experimenters were generally not blind to fly genotype (blinding is not typically used in this type of experiment because analysis is automated and not subject to experimenter bias). Behavioral experiments were performed by one individual and analyzed by a different individual blind to genotype.

Reporting for specific materials, systems and methods

We require information from authors about some types of materials, experimental systems and methods used in many studies. Here, indicate whether each material, system or method listed is relevant to your study. If you are not sure if a list item applies to your research, read the appropriate section before selecting a response.

Materials & experimental systems

n/a	Involved in the study
☒	☐ Antibodies
☒	☐ Eukaryotic cell lines
☒	☐ Palaeontology
☐	☒ Animals and other organisms
☒	☐ Human research participants
☒	☐ Clinical data

Methods

n/a	Involved in the study
☒	☐ ChIP-seq
☒	☐ Flow cytometry
☒	☐ MRI-based neuroimaging

Animals and other organisms

Policy information about studies involving animals; ARRIVE guidelines recommended for reporting animal research

Laboratory animals	For this study we used 5-7 day old male Drosophila and the following strains were used: Canton-special, UAS-Kir2.1, UAS-DTI, HC-Gal4, HC-LexA, AC-Gal4, trpA11, Df trpa1, Df Gr28B, VT46265-Gal4, UAS-GFP, UAS-GCaMP7f, hs-FLPG5.PEST and tubP-FRT>GAL80-FRT>, and Gr28B excision 8 and excision 66 mutants.
Wild animals	This study did not involve wild animals.
Field-collected samples	This study did not involve samples collected in the field.
Ethics oversight	Ethics approval was not required for experiments on invertebrates.

Note that full information on the approval of the study protocol must also be provided in the manuscript.
